# Why Do Floral Perfumes Become Different? Region-Specific Selection on Floral Scent in a Terrestrial Orchid

**DOI:** 10.1371/journal.pone.0147975

**Published:** 2016-02-17

**Authors:** Karin Gross, Mimi Sun, Florian P. Schiestl

**Affiliations:** Institute of Systematic Botany, University of Zurich, Zollikerstrasse 107, CH-8008, Zurich, Switzerland; University of Naples Federico II, ITALY

## Abstract

Geographically structured phenotypic selection can lead to adaptive divergence. However, in flowering plants, such divergent selection has rarely been shown, and selection on floral signals is generally little understood. In this study, we measured phenotypic selection on display size, floral color, and floral scent in four lowland and four mountain populations of the nectar-rewarding terrestrial orchid *Gymnadenia odoratissima* in two years. We also quantified population differences in these traits and pollinator community composition. Our results show positive selection on display size and positive, negative, or absence of selection on different scent compounds and floral color. Selection on the main scent compounds was consistently stronger in the lowlands than in the mountains, and lowland plants emitted higher amounts of most of these compounds. Pollinator community composition also differed between regions, suggesting different pollinators select for differences in floral volatiles. Overall, our study is the first to document consistent regional differences in selection on floral scent, suggesting this pattern of selection is one of the evolutionary forces contributing to regional divergence in floral chemical signaling.

## Introduction

One of the most intriguing characteristics of angiosperms is their striking floral diversity. Floral traits like size, shape, color, and scent act as visual or olfactory signals attracting pollinators [1, 2], but also impact on plant-herbivore interactions [3]. Adaptation to specific pollinators plays an important role in the evolution of flower diversity in angiosperms [4–6]. Pollinators show preferences towards different floral signals [7–10]. Therefore, pollinators can select for floral signal divergence [11–13] and facilitate diversification in floral signals within and between plant species [2].

At the intraspecific level, geographically structured divergence in floral traits is common [14]. Also, differences in pollinator communities in widely distributed plant species, particularly along altitudinal gradients, are commonly found [15–19]. Regional differences in pollinator communities, can impose divergent selection, resulting in complex geographical selection mosaics [20–23]. In several plant species, studies suggest that divergence in floral morphology such as spur length or the extent of herkogamy, as well as in floral color results from regional differences in pollinator communities (e.g., [9, 24–27]. However, in most studies, evidence for divergent natural selection as the cause for floral-trait divergence is not compelling, and trait divergence could also result from phenotypic plasticity or genetic drift [14]. In particular, the causes for regional divergence in floral signals, particularly floral scent, are not well understood [28–32].

Floral scent as well as floral color are key traits for plant-insect interactions [2]. Through several functional studies we know that floral scent, which is usually a complex bouquet of volatile organic compounds (VOCs), can have different functions ranging from attraction of pollinators to deterring antagonists [33, 34]. Floral scent often shows considerable variation both regionally and between plant species [1]. Despite the undisputed importance of floral scent for plant reproductive success, few studies on floral trait evolution have incorporated this trait. As a consequence, we know little about the relative importance of scent in mediating plant-pollinator interactions and its role in adaptive plant diversification. Floral color, on the other hand, is a relatively well investigated sensory modality and plays a role in many aspects of plant-pollinator interactions [35–37]. Thus, selection on color mediated by the pollinator’s preferences can be expected and was detected in some [25, 38] but not in all past studies focusing on this floral signal [39, 40].

In the present study, we measured phenotypic selection on floral signals in several lowland and mountain populations in the orchid *Gymnadenia odoratissima*; most of these populations were investigated in two consecutive flowering seasons. In total, 1028 plants were analyzed. *Gymnadenia odoratissima* grows over a wide altitudinal range, from lowlands to the alpine zone [41]. It produces nectar in a short floral spur and has a functionally specialized pollination system (sensu [42], with a range of primarily lepidopteran pollinators [33, 43, 44]. In Switzerland, where we conducted the study, *G*. *odoratissima* forms locally abundant populations, making it a viable system to investigate geographically structured differences in phenotypic selection in relationship to differences in pollinator community composition. Floral signals differ considerably between lowland and mountain *G*. *odoratissima* plants [44]. In our study we investigated phenotypic selection on floral signals, and in particular patterns of divergent selection by addressing the following questions: (i) Which floral signals are under phenotypic selection in lowland and mountain populations? (ii) Does selection on floral signals differ consistently between altitudinal regions and within regions among populations? (iii) Does spatial and/or temporal variation in selection differ between different visual and olfactory floral signals? (iv) How do pollinator communities and floral signals differ between regions and/or between populations within regions? Does herbivory contribute to selection?

## Materials and Methods

### Study species and populations

The terrestrial orchid *Gymnadenia odoratissima* (L.) L.C.M. Rich. (common name: Short-Spurred Fragrant Orchid) has a geographic distribution restricted to the temperate zone of Europe [45, 46] and grows almost exclusively on calcareous soil from the lowlands to the alpine zone [41]. In Switzerland, where we conducted the study, *G*. *odoratissima* occurs within an altitudinal range from 300 to 2400 m a.s.l. and grows in locally abundant populations. Like many orchids, *G*. *odoratissima* is perennial but does not flower every year. In flowering plants, a single inflorescence consisting of approximately 10–140 flowers is produced. Floral color ranges from dark purple to pale pink in the lowlands and from pink to white in the mountains. The species is self-compatible but largely outcrossing and the strong, sweet floral scent is important to attract pollinators [33]. Six scent compounds (mostly aromatics) were shown to elicit electrophysiological (EAD) responses in olfactory neurons of pollinator insects, and one of these compounds (phenylacetaldehyde) was also found to attract pollinators in the field [33]. We conducted the present study in eight natural populations–four lowland populations in north-eastern Switzerland (Döttingen, Remigen, Linn, and Rossweid; 500–650 m a.s.l.) and four mountain populations in south-eastern Switzerland (Schatzalp, Münstertal, Albulapass, and Corviglia; 1800–2250 m a.s.l.)–and between 2010 and 2012 (S1 Table). The ‘Departement Bau, Verkehr und Umwelt; Abteilung Landschaft und Gewässer; Sektion Natur und Landschaft’ from Kanton Aargau, Switzerland, the ‘Amt für Landschaft und Natur; Fachstelle Naturschutz’ from Kanton Zurich, Switzerland, and the ‘Amt für Natur und Umwelt; Abteilung Natur und Landschaft’ from the Kanton Grisons, Switzerland provided collection permits.

### Measurement of floral signals

Our study was conducted in three lowland and three mountain populations in 2010, and in four lowland and three mountain populations in 2011; in three of these populations in the lowlands and in two in the mountains, we investigated plants in both years (S1 Table). When most plants were in full flower in a population (lowland: end of June to mid-July, mountain: mid-July to mid-August), we marked individual plants along a transect. In each population and year, 100 plants were marked, except in the lowland population Remigen (60 plants) and the mountain population Schatzalp (99 plants) in 2011 (S1 Table). Two to four days were needed to measure the floral signals of all marked plants in a population.

We measured plant height (ground to uppermost flower) and inflorescence length (calculated as the difference between plant height and stem length [ground to lowermost flower]) to the nearest centimeter using a measuring tape. In addition, we counted the total number of flowers. Plant height, inflorescence length, and the total number of flowers were used as three measures of display size in further analyses.

We collected floral scent for 30 min at some time between 9:00 a.m. and 7:00 p.m. on days without rain using headspace sorption, a non-invasive method that does not damage the plant from which scent is collected. Due to the large number of plants (*n* > 1000 plants), and the length of time required for scent collection, it was not possible to collect scent at the same time for all plants; changes in scent emission during the day are, however, negligible in *G*. *odoratissima* (unpublished data). We enclosed the inflorescence of each individual in an oven bag (Nalophan®) tied closed with short pieces of florist wire. A small glass tube, filled with approximately 20 mg 80/100 mesh Tenax® absorbent powder (Supelco, Bellefonte, PA, USA; called “filter” hereafter), was inserted into each bag. The filter was connected to a battery-operated vacuum pump (PAS-500 Micro Air Sampler, Spectrex, Redwood City, CA, USA) using a silicone tube. Air was vacuum pumped out of the bag through the filter at a rate of 150 ml min^-1^, trapping the floral volatiles on the Tenax® adsorbent. We collected air from one to two empty bags per population to control for contaminants from the surrounding air. After scent collection, we wrapped the filters’ ends with PTFE (Teflon®) thread seal tape and packed each individual filter in aluminum foil or in a small glass vial. Filters were stored in a -30°C freezer until analysis. Samples were analyzed by gas chromatography with mass selective detection (GC-MSD). A thermal desorption system (TDS3, Gerstel, Mühlheim an der Ruhr, Germany; solvent vent mode (splitless) with a cold injection system (CIS4, Gerstel, Mühlheim an der Ruhr, Germany) was used to inject samples into an Agilent GC 6890N gas chromatograph (Agilent Technologies, Palo Alto, CA, USA), which was equipped with an HP-5 column (0.25 mm diameter, 0.32 μm film thickness, 30 m length), and helium was used as carrier gas at a flow rate of 1.9 ml min^-1^. The TDS temperature was programmed to rise from 30°C (0.5 min hold) to 240°C (1 min hold) at 60°C min^-1^ for thermal desorption; the CIS temperature was -150°C during thermal desorption and was programmed to rise from -150°C (0.5 min hold) to 150°C at 16°C s^-1^ and from 150°C to 250°C (0.5 min hold) at 12°C s^-1^ for injection. The GC oven temperature was programmed to rise from 50°C to 230°C at 8°C min^-1^. The GC was connected to an Agilent MSD 5975 mass selective detector (Agilent Technologies, Palo Alto, CA, USA). Compounds were identified by comparing obtained mass spectra with those from the NIST spectral reference database (NIST 05) implemented in the ChemStation Enhanced Data Analysis program (G1701EA E.02.02 MSD Productivity ChemStation Software, Agilent Technologies, Germany). Compound identification was verified by comparing obtained mass spectra with those of synthetic standards of all compounds. One or two concentrations of these synthetic standard compounds were analyzed to obtain calibration curves using the peak area of a compound-specific qualifier ion. The calibration curves were employed to convert the peak areas of the compound-specific qualifier ions in the *G*. *odoratissima* samples into nanograms using the ChemStation program. We manually double-checked all samples and compounds and, if necessary, manually integrated the peak area. For analysis, we calculated compound amounts in nanograms per liter sampled air per inflorescence. We included a compound as floral scent compound when it met the following criteria: (i) its median concentration for air controls was lower than 80% of its mean concentration for plant samples in the corresponding population for at least one year, and (ii) its mean concentration for plant samples was higher than 0.5 ng l^-1^ per inflorescence in both years. Applying these criteria resulted in a list of 22 floral scent compounds.

Floral color was only measured in 2011. Due to time constraints during field work, floral color was quantified as categorical intensity rather than by spectrophotometry. We cut off two open flowers per individual (one from the bottom and another from the top of the inflorescence), placed them on a white paper, and photographed them. We determined five flower photographs as standards according to 1 “white”, 2 “pinkish white”, 3 “light pink”, 4 “pink”, and 5 “purple”. The color of all other photographed flowers was classified on a computer screen using these standards. This simplified color assessment is justifiable in *G*. *odoratissima* as spectral reflectance of its flowers is restricted to the wavelength range visible for the human eye, and floral color differences primarily result from differences in the relative spectral reflectance in the wavelength range between 488 nm and 636 nm [44].

### Female reproductive success

Upon maturation, number of fruits generated by the plants of which floral signals were measured was counted. Some plants or labels were missing due to browsing herbivores and mowing. Fitness was estimated by calculating relative female reproductive success (fRS) as the number of fruits produced by an individual divided by the mean number of fruits produced by all the marked plants in the same population and year. In addition, the proportional fRS was calculated as the number of fruits divided by the total number of flowers for each individual plant. Female reproductive success generally differed between populations both in the lowland and the mountain region, but it generally did not differ between altitudinal regions (S1 Text; S2 Table). In our study, it was not feasible to assess male reproductive success, as this would have required multiple scoring of pollinia removal rates during the flowering season.

### Pollinator (pollen) limitation

A prerequisite for pollinator-mediated selection through the female fitness component (production of seeds) is the limitation of a plant’s reproductive success by incoming pollen through pollinators (here called “pollinator limitation”) rather than by resources. To quantify pollinator limitation, we conducted a pollination experiment in the field with three treatments: hand cross pollination, pollinator exclusion, and open pollination as a control. For the hand-pollination treatment, we supplementally hand-pollinated all the flowers of 4–10 plants per population in the lowland population Döttingen and the mountain populations Schatzalp and Albulapass in 2010 and in the lowland populations Döttingen, Remigen, Linn, and Rossweid and the mountain populations Schatzalp, Münstertal, and Corviglia in 2011 (S1 Table). We pollinated flowers with pollinaria collected from conspecific plants at least five meters away, using wooden toothpicks or tweezers. The hand-pollinated plants were marked and bagged with fine-meshed wire insect nets (tesa® AG, Hamburg, Germany) to prevent further pollinator visitations. For the pollinator-exclusion treatment, we bagged four plants in the lowland population Döttingen and in the mountain population Albulapass in 2010 to prevent pollinator visitations. Plants used for floral signal quantification (see above) served as the untreated open-pollination control plants. When fruits were mature, we counted the total number of flowers and fruits produced per inflorescence to calculate the proportional fRS. For each population and year, we calculated the extent of pollen limitation as 1 –(mean proportional fRS of open-pollinated control plants/mean proportional fRS of hand-pollinated plants) [47].

### Pollinator community composition and floral herbivory

We assessed pollinator community composition in all populations where we quantified floral signals. Pollinators were observed and caught between 7:00 a.m. and 11:00 p.m. between 29 June and 09 August in 2010, 2011, and 2012 by slowly walking through the populations. Insects were considered pollinators if they probed *G*. *odoratissima* flowers and either removed pollinaria or, in case pollinaria were already removed, exhibited behavior likely leading to pollinaria removal. Additionally, insects that rested on *G*. *odoratissima* inflorescences and carried pollinaria of the size of *G*. *odoratissima* pollinaria were considered pollinators. For later identification, pollinators were caught using hand nets, transferred to individual plastic tubes and stored at -30°C until preparation; alternatively, we photographed the pollinators in the field. When at least one individual of a pollinator species had previously been caught or photographed, we only recorded the observation. For each observed pollinator, we documented the population, date, and observation time and noted whether the pollinator carried pollinaria on its proboscis. Specimens could not always be identified to the species or genus level; thus, for statistical analysis, we used the family level for Lepidoptera and order level for other insects. We only considered populations where we caught and/or observed pollinators on at least two different days; these were the lowland populations Döttingen (5 d), Remigen (5 d), Linn (3 d), and Rossweid (2 d) and the mountain populations Schatzalp (12 d), Albulapass (2 d), and Corviglia (2 d). Median total observation time per population did not differ between the lowland (18.00 h) and mountain (19.25 h) region (Kruskal-Wallis test: d.f. = 1, test statistics = 0.000, *P* = 1), but differed considerably between populations within regions (lowland region: 27.00 h in Döttingen, 19.75 h in Remigen, 14.50 h in Linn, 16.25 h in Rossweid; mountain region: 37.50 h in Schatzalp, 2.00 h in Albulapass, 19.25 h in Corviglia). These differences were accounted for by calculating the total number of pollinators and number of pollinator families/orders caught and observed per hour. The number of pollinators per hour served as a measure of visitation rate and the number of different pollinator families/orders per hour as a conservative measure of pollinator richness. The similarity of pollinator communities between populations and altitudinal regions was quantified by computing the Bray-Curtis dissimilarity as (Σ (|*y*_*i1*_ –*y*_*i2*_|)) / (Σ (*y*_*i1*_ + *y*_*i2*_) [48] between all days and populations when pollinators were caught and observed using the package *ecodist* (version 1.2.7 [49]) in the statistical software program R (version 3.0.1 [50]).

As we observed some floral herbivory in the form of eaten flowers and aphid infestation, we quantified the magnitude of floral herbivory for all marked plants on the day we measured floral signals as described in the S1 Text. Floral herbivory was generally low in our study populations (S1 Fig). It generally differed between populations in both altitudinal regions, but was not different between the lowland and the mountain region (S1 Fig).

### Statistical analyses

For most statistical analyses, we used SPSS 20.0.0.0 for Windows (IBM SPSS Statistics, IBM Corp., Released 2011, Armonk, NY, USA). R [50] was used for the remaining statistical analyses (as stated in the text) due to its suitability for specific analyses.

#### Phenotypic selection analyses

Display size and floral scent were measured in 2010 and 2011, and floral color was measured only in 2011. Therefore, phenotypic selection analyses were conducted in two ways (i) in a two-year data set (2010 and 2011), containing display size and floral scent, and (ii) in a one-year data set (2011), which included display size, floral scent, and floral color. Results from the two analyses did not differ for display size and floral scent; therefore we describe the statistical analyses and report results for the two-year data set and provide the results for the analyses on the 2011 data set in S2 and S3 Figs.

A principal component analysis (PCA) was performed on all floral display and floral scent traits as well as all populations and both study years to reduce the number of variables and convert the potentially correlated variables into linearly uncorrelated principal components (PCs). The PCA was conducted with traits standardized to a mean of 0 and a standard deviation (SD) of 1 for each population. We extracted principal components (PCs) with an eigenvalue > 1 using varimax rotation (an orthogonal rotation minimizing the number of variables with high loadings on each PC, which simplifies the interpretation of the PCs). This procedure resulted in seven PCs: one “display size PC” (PC3) and six “floral scent PCs” (PC1, PC2, PC4, PC5, PC6, PC7) explaining 71.8% of the total variance (S3 Table). Trait-value distributions of the PCs are shown in S4 Fig. These PCs were used as explanatory variables in the phenotypic selection analyses. Preliminary analyses did not indicate quadratic selection; therefore we assessed only linear selection in the final analysis. To determine which floral signals were under selection, we estimated selection gradients *β* on each PC, using linear multiple regression analyses in R [50, 51]. Relative fRS was used as response variable and PCs as explanatory variables. To assess whether selection on PCs differed between altitudinal regions and/or years, we used a linear mixed model using the package *lme4* [52] in R [50]. The model included relative fRS as the response variable, PCs as covariates, altitudinal region, year, interactions between each PC and altitudinal region, and interactions between each PC and year as fixed factors, and population nested within altitudinal region as random factor. In addition, we conducted linear models in R [50] to test for differences in selection among populations within altitudinal regions and between years. In these models, we included relative fRS as the response variable, PCs as covariates, and population, year, interactions between each PC and population, and interactions between each PC and year as fixed factors.

#### Pollinator limitation

Pollinator limitation in fruit production was tested by comparing the proportional fRS between the hand-pollination and the open-pollination treatment using a Mann-Whitney U test for each year and population. Similarly, we compared the proportional fRS between the pollinator-exclusion and the open-pollination treatment to test for pollinator dependency.

#### Pollinator community composition

Differences in pollinator community composition were tested by conducting PERMANOVAs (Permutational Multivariate Analysis of Variance, an ANOVA using Bray-Curtis dissimilarity data and permutation tests with pseudo-*F* ratios) using the package *vegan* (version 2.0–9 [53]) in R [50]. To test for differences between the lowland and the mountain region, populations were used as replicates and region was used as fixed factor; to test for differences between populations within regions, days were used as replicates and population was used as fixed factor. We generated a non-metric multidimensional scaling (NMDS) plot based on Bray-Curtis dissimilarities employing the PROXSCAL procedure with ordinal proximity transformation and Torgerson as initial configuration to visualize differences. We conducted SIMPER (Similarity Percentages) analyses based on Bray-Curtis dissimilarities to determine which pollinators characterized the differences between altitudinal regions and between populations within altitudinal regions using the package *vegan* (version 2.0–9 [53]) in R [50]. Differences in pollinator visitation rates and pollinator richness were analyzed with Kruskal-Wallis tests. To test for differences between the altitudinal regions, populations were used as replicates; to test for differences between populations within regions, days were used as replicates.

#### Differences in floral signals

To assess geographic differences in floral signals, we conducted general linear models with altitudinal region, year, and population nested within region as explanatory variables. For this analyses, all trait values were ln(x + 1) transformed.

## Results

### Phenotypic selection

#### Phenotypic selection at the regional (altitudinal) level

**Fig 1:**
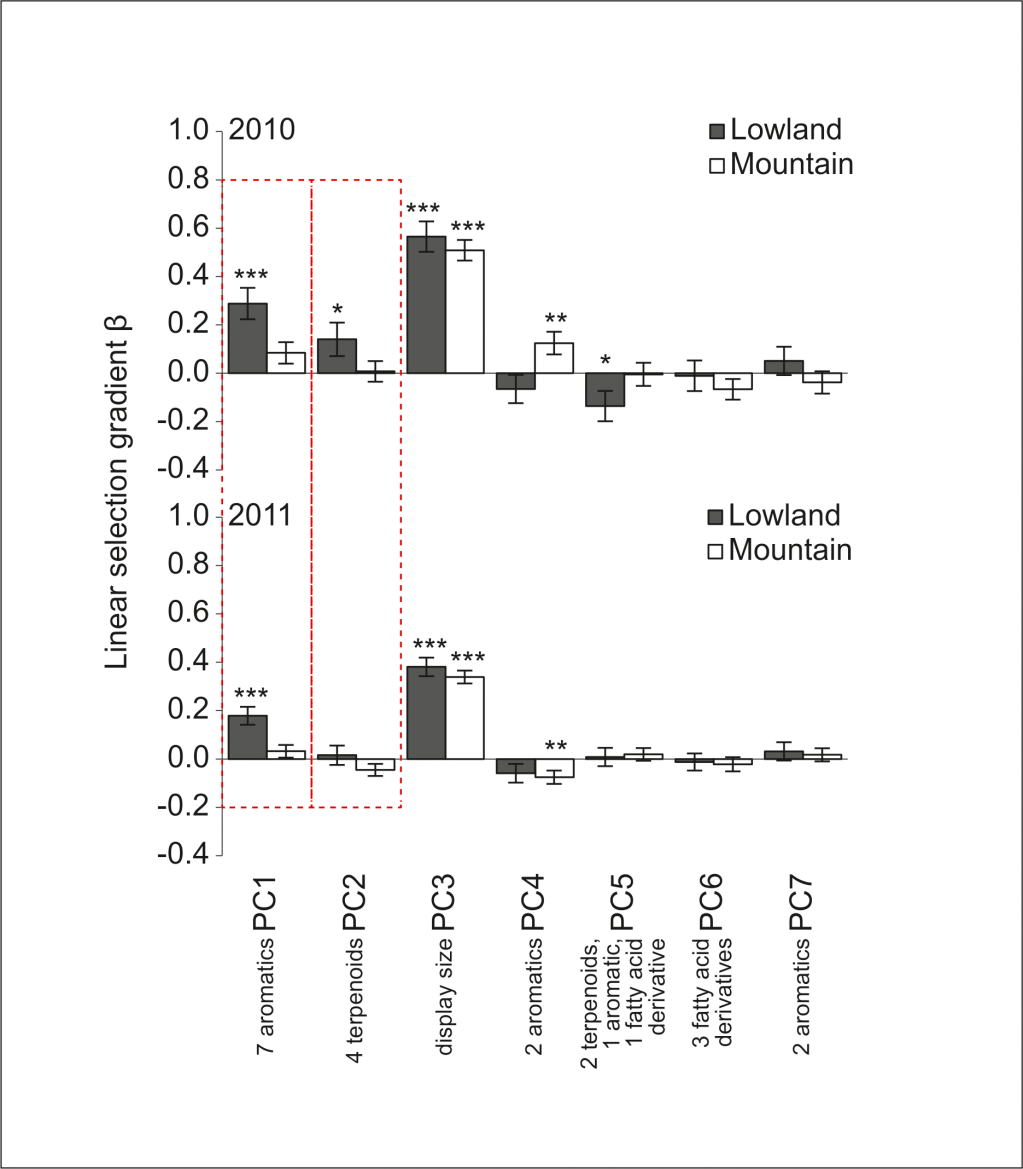
Linear selection gradients *β* ± SE for principal components (PCs) in the lowland and mountain regions in *Gymnadenia odoratissima*. Two floral scent PCs (PC1 and PC2, marked with red dotted quadrangles) show consistent spatial differences in selection indicated by significant differences between regions but not between years (see Table 1 for details). The x-axis legend gives a short summary of the floral signals loading primarily on each PC (for details, see S3 Table). Significances of linear selection gradients are indicated above the bars: ***P < 0.001, **P < 0.01, *P < 0.05. Sample sizes are 2010: *n*_lowland_ = 253 (three populations), *n*_mountain_ = 212 (three populations); 2011: *n*_lowland_ = 312 (four populations), *n*_mountain_ = 251 (three populations).

In our phenotypic selection analysis we found several traits under directional selection, and consistent differences in selection between the regions in some traits. The strongest selection was found on display size (principal component [PC] 3), which was under positive selection in both regions and in both years, with selection being stronger in 2010 than in 2011 (Fig 1, Table 1). Three out of six floral scent PCs (PC1, PC2, PC4) were under positive selection and two out of six floral scent PCs (PC4, PC5) were under negative selection in at least one region and year, with selection on floral scent PC5 differing between 2010 and 2011. Floral color and two aromatic compounds (PC7A, named “floral color and scent PC” hereafter; assessed in a separate analysis that only contained the 2011 data set; see S4 Table), were under negative selection in both altitudinal regions (S2 Fig).

**Table 1:** Principal components (PCs, with the floral signals with highest loadings on them) and differences in selection gradients between regions and years. This table shows statistics of a linear mixed model testing for differences in directional selection on PCs between altitudinal regions and years. For PC1 and PC2, significant interactions between PC and region, but not between PC and year were found, indicating consistent differences in selection between regions. For PC3 and PC5, selection differed between years rather than between regions (see also Fig 1).

	PC x Region		PC x Year	
Principle components (traits with highest loadings)	χ^2^_1_	*P*	χ^2^_1_	*P*
PC1 (7 aromatics: benzaldehyde, phenylacetaldehyde, benzyl acetate, 1-phenyl-1,2-propanedione, phenylethylacetate, 1-phenyl-2,3-butanedione, eugenol)	13.757	**< 0.001**	3.816	0.051
PC2 (4 terpenoids: α-pinene, sabinene, β-pinene, limonene)	5.450	**0.019**	3.615	0.057
PC3 (plant height, inflorescence length, number of flowers)	1.744	0.187	14.764	**< 0.001**
PC4 (2 aromatics: benzyl alcohol, phenylethyl alcohol)	2.930	0.087	2.867	0.090
PC5 (1 aromatic: styrene; 2 terpenoids: 6-methyl-5-hepten-2-one, geranyl acetone; 1 fatty acid derivative: heptanal)	1.927	0.165	5.117	**0.024**
PC6 (3 fatty acid derivatives: (Z)-3-hexen-1-ol, (Z)-3-hexenyl acetate, hexyl acetate)	0.462	0.497	0.293	0.589
PC7 (2 aromatics: methyl eugenol, benzyl benzoate)	1.683	0.195	0.030	0.863

Note: Floral signals exhibiting highest loadings on each PC are listed in brackets. For more details, see S3 Table.

Consistent differences in selection between the regions (divergent selection) were only found for floral scent (Fig 1; Table 1). Selection on floral scent PC1 and PC2 was stronger in the lowland compared to the mountain region in both years (Fig 1; Table 1). In contrast, selection on display size (PC3; Table 1) and the floral color and scent PC (PC7A; S2 Fig) did not differ between the regions.

#### Phenotypic selection at the population level

**Fig 2:**
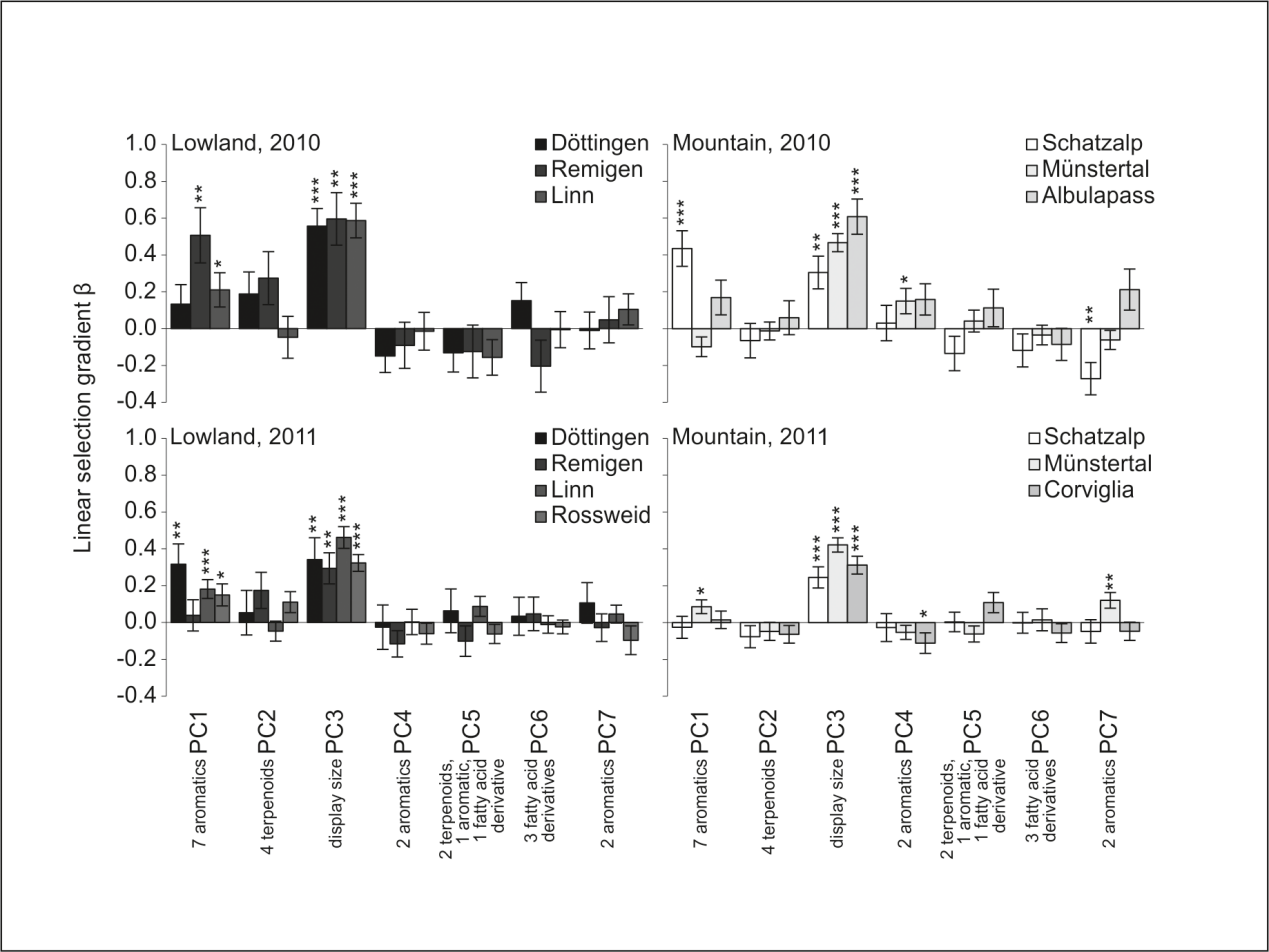
Linear selection gradients *β* ± SE for principal components (PCs) in lowland (left) and mountain (right) populations in *Gymnadenia odoratissima*. Significances of the linear selection gradients *β* are indicated above the bars: ***P < 0.001, **P < 0.01, *P < 0.05. For details of PC loadings, see S3 Table. 2010: *n*_Döttingen_ = 73, *n*_Remigen_ = 88, *n*_Linn_ = 92, *n*_Schatzalp_ = 47, *n*_Münstertal_ = 96, *n*_Albulapass_ = 69; 2011: *n*_Döttingen_ = 92, *n*_Remigen_ = 56, *n*_Linn_ = 92, *n*_Rossweid_ = 72, *n*_Schatzalp_ = 75, *n*_Münstertal_ = 94, *n*_Corviglia_ = 82.

At the population level we found significant directional selection on several traits, but no temporally consistent differences in selection between populations (Fig 2). The most pronounced selection was again found on display size (PC3), which was under positive selection in all populations and in both years. Selection on floral scent varied greatly among populations and between years. In the lowlands, only floral scent PC1 was under positive selection in some populations for both years. In the mountains, three out of six floral scent PCs (PC1, PC4, and PC7) were under positive or negative selection in at least one population and year. Floral color (PC7A) was under negative selection in only one mountain population (S3 Fig).

Selection on none of the PCs differed consistently between populations (Fig 2; Table 2). Selection on display size (PC3) differed between mountain populations, but also differed temporally in the lowland populations, in being stronger in 2010 than in 2011. Floral scent PC7 differed between populations as well as between years in the mountain region. Selection on the floral color and scent PC (PC7A) did not differ between populations (S3 Fig).

**Table 2:** Differences in selection gradients between populations and years. The table shows statistics of a linear model testing for differences in directional selection on principal components (PCs) between populations and years in the lowland and the mountain regions.

	Lowland				Mountain			
	PC x Population		PC x Year		PC x Population		PC x Year	
PC	*F*_3_	*P*	*F*_1_	*P*	*F*_3_	*P*	*F*_1_	*P*
PC1	0.037	0.772	1.383	0.240	2.164	0.092	0.398	0.528
PC2	1.819	0.143	1.271	0.260	0.390	0.761	0.352	0.554
PC3	0.225	0.879	5.547	**0.019**	3.024	**0.029**	1.821	0.178
PC4	0.492	0.688	0.190	0.664	0.735	0.531	3.133	0.077
PC5	0.361	0.782	3.583	0.059	2.329	0.074	0.040	0.841
PC6	1.157	0.326	0.001	0.978	0.425	0.735	1.588	0.208
PC7	0.278	0.841	0.027	0.868	6.690	**< 0.001**	7.389	**0.007**

Note: For a list of floral signals exhibiting highest loadings on each PC, see Table 1. For details, see S3 Table.

### Pollinator limitation

Pollinator limitation was strong in most populations and ranged from 0.38 to 0.63 (median = 0.51) in 2010 and from 0.10 to 0.76 (median = 0.42) in 2011. The proportional fRS was significantly higher in hand-pollinated compared with open-pollinated plants in two of three populations in 2010 and in six of seven populations in 2011 (S5 Table). Moreover, we found low figures of spontaneous self-pollination; in plants from which pollinators were excluded the proportional fRS was very low (Döttingen: 0.00–1.59%; Albulapass: 0.00–3.85%), and significantly lower than in open-pollinated plants (Döttingen: *n*_open_ = 75, *n*_bagged_ = 4, *z* = 2.886, *P* = 0.004; Albulapass *n*_open_ = 85, *n*_bagged_ = 4, *z* = 2.615, *P* = 0.009). Thus, *G*. *odoratissima* plants largely depend on pollinators to set fruits.

### Differences in pollinator community composition

**Fig 3:**
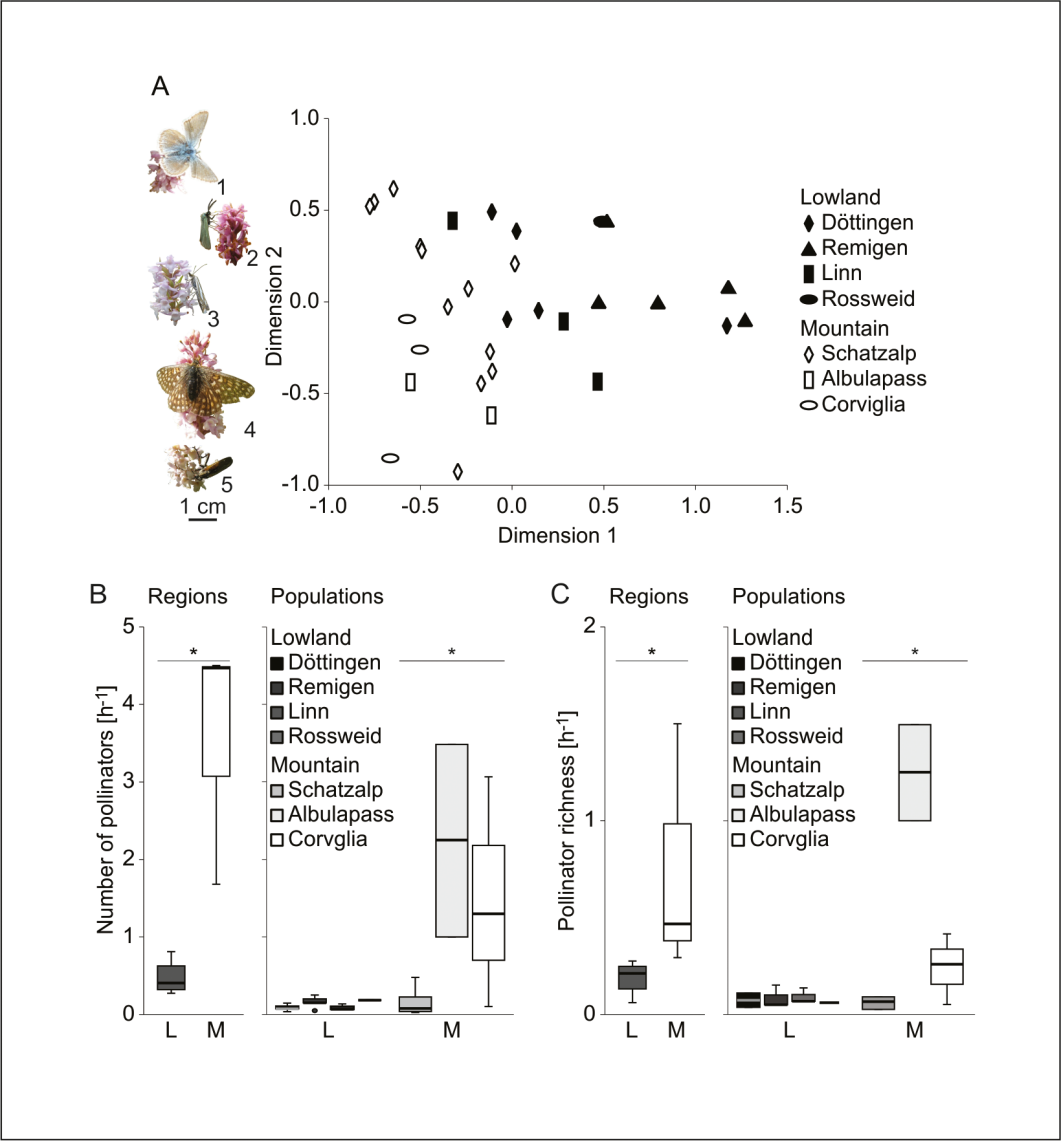
Differences in pollinator community composition in lowland and mountain populations of *Gymnadenia odoratissima*. (A) Non-metric multidimensional scaling (NMDS) of pollinator community composition in four lowland and three mountain populations. Pollinators shown to the left of the plot represent the five taxa (“1” Lycaenidae, “2” Zygaenidae, “3” Crambidae, “4” Nymphalidae, “5” Diptera), that contributed most to the community differences between the lowland and mountain region and were all more common in the mountain region. Boxplots show (B) visitation rate and (C) pollinator richness (number of pollinator families/orders per hour) compared between regions (left) and populations (right). Mountain populations were significantly more rich in pollinators and showed higher pollinator abundances (*P < 0.05).

We found a relatively diverse community of pollinators, which differed between the regions. We identified a total of 196 pollinators from three insect orders: primarily Lepidoptera (13 families), some Diptera (all identified as Empididae), and few Coleoptera (S6 Table). Pollinator community composition differed significantly between altitudinal regions (Fig 3; pseudo-*F*_1,5_ = 3.289, *P* = 0.030). Lycaenidae, Zygaenidae, Crambidae, Nymphalidae, and Empididae (Diptera) contributed most to the differences between lowland and mountain pollinator communities and were more common in the mountain region, with Zygaenidae and Diptera being exclusively found in the mountain populations (Fig 3A; S7 Table). At the population level, pollinator community composition differed significantly between mountain populations (pseudo-*F*_2,14_ = 2.147, *P* = 0.008) and, though still significant, to a lesser extent between lowland populations (pseudo-*F*_3,11_ = 1.851, *P* = 0.028; Fig 3A). Noctuidae, Hesperiidae, Nymphalidae, Pyralidae, Pterophoridae, Pyralidae, and Coleoptera were the primary contributors to differences between lowland pollinator communities, and Zygaenidae, Lycaenidae, Crambidae, and Nymphalidae primarily contributed to differences between mountain pollinator communities (S7 Table). Moreover, pollinator visitation rate and pollinator richness were significantly higher in the mountains compared to the lowlands and significantly differed between mountain but not between lowland populations (Fig 3B and 3C).

### Trait differences

Most traits differed between regions, but also between populations and years (S8 Table). Lowland plants exhibited larger displays and darker flowers compared to mountain plants. For floral scent, the amount of most compounds differed between lowland and mountain plants; the total amount of scent, however, was not different between regions.

## Discussion

In flowering plants, regional divergence in floral traits is widespread. A possible explanation is consistent region-specific (divergent) selection. Our large-scale phenotypic selection study in a terrestrial orchid supports this scenario by documenting consistent divergent selection in mountain and lowland regions. This pattern of divergent selection was, however, only found for floral olfactory signals. Our study is the first that documents region-specific selection on floral scent.

Geographically structured selection on floral signals can thus result from consistent spatial differences in preferences of (the most efficient) pollinators towards floral signals [7–10]. Additionally, antagonists, such as florivores, or abiotic selection agents, can counteract or reinforce pollinator-mediated selection [3, 5, 54, 55]. Although our study was not specifically designed to test for the agent of locally variable selection, our results suggest that phenotypic selection in *G*. *odoratissima* was primarily pollinator-mediated. First, fruit set was strongly pollen limited in most populations, a prerequisite for pollinator-mediated selection through female fitness. Second, the pollinator community composition differed between altitudinal regions and between populations. Third, the extent of herbivory also differed among populations, but was generally low (S1 Fig).

Pollinator-mediated selection is expected on both floral signals and flower morphology [1, 2]. Among floral signals, selection on visual signals is better documented. For example, selection for larger displays has commonly been found [47, 56–59]. Our findings are consistent with these studies, suggesting that pollinators often prefer large displays, either because larger displays contain more reward or are better visible [60]. While some studies report selection on floral color [38, 61], others found no evidence for it [40]. In our study, weak selection for lighter-colored flowers in *G*. *odoratissima* was detected and might be imposed by nocturnal pollinators, which are known to visit *G*. *odoratissima* [33, 44]. Only few selection studies up to now have also included floral scent; nevertheless, three recent studies reported significant but compound-specific selection on floral scent [40, 55, 58]. This result is congruent with our results and suggests floral scent is often under selection mediated by biotic interactions.

Regional divergence in floral traits is common and has been documented in morphology and floral signals [9, 24, 25, 28, 30, 62, 63]. In principle, such divergence can result from phenotypic plasticity, genetic drift, or natural selection [14]. Consistent spatial variation in selection is expected to result in adaptive population divergence, if the variation in traits under selection has a heritable component [64, 65]. In our study, consistent region-specific differences in selection were evident on two floral scent PCs, which represent the major floral scent compounds. As floral scent has recently been shown to have considerable heritability [66], we can predict adaptive divergence in these scent compounds. Indeed, the stronger directional selection on PC1 in the lowlands was matched by a higher emission of five of these compounds in the lowlands (S8 Table). Six of the seven PC1-compounds (benzaldehyde, phenylacetaldehyde, 1-phenyl-2,3-butanedione, benzyl acetate, phenylethyl acetate, and eugenol) were shown earlier to elicit electrophysiological responses in pollinators of *G*. *odoratissima*, and phenylacetaldehyde attracted pollinators in the field [33]. Thus, the higher production of these volatiles may represent an adaptation to lowland pollinators. Local adaptation to pollinators was, however, only detected in mountain plants of *G*. *odoratissima* [44]. This seemingly contradictory finding may be explained by the higher pollinator richness in mountain populations. Lowland plants transferred to mountain populations may thus well attract alternative pollinators with “lowland scent”, whereas mountain plants being less attractive to lowland pollinators may suffer more under the lack of mountain-specific pollinators such as empidid flies. Nevertheless, phenotypic selection is most likely not the only evolutionary force shaping regional differences in plant traits, because many other traits including inflorescence size and floral color, for which no significant divergent selection was detected, showed significant differences between regions (S8 Table). The significant differences in plant trait between years can be explained by the fact that likely not the same plants were measured every year in the investigated populations.

In conclusion, our study suggests that spatial variation in average selection was consistent enough to create geographically structured selection, leading to regional divergence in floral traits. Our data emphasize the importance of measuring phenotypic selection in multiple populations and in different flowering seasons to capture the actual selection dynamics acting within a species. Furthermore, our results provide indication that geographically structured differences in selection do not act equally on all traits, suggesting different evolutionary forces acting on different traits and possibly different evolutionary rates in different traits. Future studies should quantify phenotypic selection on floral-morphology traits affecting pollinator efficiency, such as floral spur length, to reach a more conclusive understanding of the evolution of flowers as a whole.

## Supporting Information

S1 Fig Percentage of plants experiencing floral herbivory (upper graphs) and differences in the mean (± SE) floral herbivory (lower graphs) in lowland and mountain populations of *Gymnadenia odoratissima*. Floral herbivory was quantified as (A) number of eaten flowers per inflorescence and (B) aphid load (scale from 1 [no aphids] to 6 [many aphids]). Sample sizes are indicated inside the top of the percentage bars. Whereas populations within regions differed in floral herbivory, no consistent differences in floral herbivory was found between lowland and mountain regions (***P < 0.001, **P < 0.01, *P < 0.05, ns *P* > 0.05).
(PDF)

S2 Fig Linear selection gradients *β* ± SE for principal components (PCs) in the lowland and mountain region in *Gymnadenia odoratissima* for the 2011 data set, which included floral color. Whereas several PCs showed significant selection gradients (marked with asterisks above bars), only PC1 showed significant differences between the regions (***P < 0.001, **P < 0.01, *P < 0.05). A short description of the floral signals loading primarily on each PC is given; for details, see S4 Table. According to variables loading primarily on PCs, PC1A corresponds to PC1 in Fig 1, PC2A to PC2, PC3A to PC5, PC4A to PC3, PC5A to PC6, PC6A to PC7, and PC7A to PC4 except that floral color additionally loaded primarily on PC7A. *n*_lowland_ = 312 (four populations), *n*_mountain_ = 251 (three populations).
(PDF)

S3 Fig Linear selection gradients *β* ± SE for principal components (PCs) in lowland (left) and mountain (right) populations in *Gymnadenia odoratissima* for the 2011 data set, which included floral color. Several PCs showed significant selection gradients and PC6A was significantly different among mountain populations (***P < 0.001, **P < 0.01, *P < 0.05). A short description of the floral signals loading primarily on each PC is given; for details, see S4 Table. According to variables loading primarily on PCs, PC1A corresponds to PC1 in Fig 2, PC2A to PC2, PC3A to PC5, PC4A to PC3, PC5A to PC6, PC6A to PC7, and PC7A to PC4 except that floral color additionally loaded primarily on PC7A. *n*_Döttingen_ = 92, *n*_Remigen_ = 56, *n*_Linn_ = 92, *n*_Rossweid_ = 72, *n*_Schatzalp_ = 75, *n*_Münstertal_ = 94, *n*_Corviglia_ = 82.
(PDF)

S4 Fig Comparison of the trait-value distribution of the principle components (PCs) used for selection analysis in lowland (grey bars) and mountain (white bars) regions. The distributions of the PC scores are compared with superimposed normal distributions.
(PDF)

S5 Fig Correlogram of among-population Euclidean distance in floral signals and among-population Euclidean distance in selection gradients on these PCs. For the Euclidian distances in the signals, only the variables were used that exhibited the highest loadings on the principal components (PCs) used in the selection analysis. For scent PC4 and PC5, a significant association between trait differences and selection differences was found (Mantel test statistics with 1000 permutations in all tests). *n* = 5 populations (three lowland and two mountain populations). For details on PCs, see Table 1 and S3 Table.
(PDF)

S1 Table Geographic locations of the four lowland and the four mountain study-populations of *Gymnadenia odoratissima* and year(s), in which selection and floral signals was measured and hand pollinations were conducted. (PDF)

S2 Table Female reproductive success (mean ± SE) of *Gymnadenia odoratissima* plants in the four lowland and the four mountain populations and the statistical tests between lowland populations, between mountain populations, and between the two altitudinal regions. (PDF)

S3 Table Factor loadings of display size and floral scent compounds of *Gymnadenia odoratissima* plants on principal components (PCs) using the two-year data set. (PDF)

S4 Table Factor loadings of floral signals of *Gymnadenia odoratissima* on principal components (PCs) using the 2011 data set, which included also floral color. (PDF)

S5 Table Pollinator limitation in lowland and mountain populations of *Gymnadenia odoratissima* in 2010 and 2011 assessed in a pollination experiment. (PDF)

S6 Table Pollinators caught and/or observed on *Gymnadenia odoratissima* inflorescences in the four lowland populations (Döttingen, Remigen, Linn, and Rossweid) and the three mountain populations (Schatzalp, Albulapass, and Corviglia). (PDF)

S7 Table Contribution of pollinator taxa to the differences in the pollinator communities between the lowland and the mountain region as well as between populations within altitudinal regions of *Gymnadenia odoratissima* using SIMPER (Similarity Percentages) analyses. (PDF)

S8 Table Differences in floral traits between lowland and mountain plants. (PDF)

S9 Table Median (minimum-maximum) coefficient of variation for the three floral-signal groups of *Gymnadenia odoratissima* plants. (PDF)

S1 Text (PDF)

## References

[1] Raguso RA. Start making scents: the challenge of integrating chemistry into pollination ecology. Entomol Exp Appl. 2008;128(1):196–207. 10.1111/j.1570-7458.2008.00683.x .

[2] Schiestl FP, Johnson SD. Pollinator-mediated evolution of floral signals. Trends Ecol Evol. 2013;28(5):307–15. 10.1016/j.tree.2013.01.019 . 23480953

[3] Strauss SY, Whittall JB. Non-pollinator agents of selection on floral traits In: Harder LD, Barrett SCH, editors. Ecology and evolution of flowers. Oxford: Oxford University Press; 2006 p. 120–38.

[4] Grant V. Pollination systems as isolating mechanisms in angiosperms. Evolution. 1949;3(1):82–97. 10.2307/2405454 . 18115119

[5] Harder LD, Johnson SD. Darwin's beautiful contrivances: evolutionary and functional evidence for floral adaptation. New Phytol. 2009;183(3):530–45. 10.1111/j.1469-8137.2009.02914.x . 19552694

[6] van der Niet T, Johnson SD. Phylogenetic evidence for pollinator-driven diversification of angiosperms. Trends Ecol Evol. 2012;27(6):353–61. 10.1016/j.tree.2012.02.002 . 22445687

[7] Schemske DW, Bradshaw HD Jr.. Pollinator preference and the evolution of floral traits in monkeyflowers (*Mimulus*). Proc Natl Acad Sci USA. 1999;96(21):11910–5. 10.1073/pnas.96.21.11910 . 10518550 PMC18386

[8] Vereecken NJ, Cozzolino S, Schiestl FP. Hybrid floral scent novelty drives pollinator shift in sexually deceptive orchids. BMC Evol Biol. 2010;10 10.1186/1471-2148-10-103 . PMC2875231 20409296

[9] Newman E, Anderson B, Johnson SD. Flower colour adaptation in a mimetic orchid. Proc R Soc Lond, Ser B: Biol Sci. 2012;279(1737):2309–13. 10.1098/rspb.2011.2375 . PMC3350669 22298842

[10] Hirota SK, Nitta K, Suyama Y, Kawakubo N, Yasumoto AA, Yahara T. Pollinator-mediated selection on flower color, flower scent and flower morphology of *Hemerocallis*: evidence from genotyping individual pollen grains on the stigma. Plos One. 2013;8(12). 10.1371/journal.pone.0085601 . PMC3871637 24376890

[11] Campbell DR, Waser NM, Meléndez-Ackerman EJ. Analyzing pollinator-mediated selection in a plant hybrid zone: hummingbird visitation patterns on three spatial scales. Am Nat. 1997;149(2):295–315. 10.1086/285991 .

[12] Gómez JM, Bosch J, Perfectti F, Fernández JD, Abdelaziz M, Camacho JPM. Spatial variation in selection on corolla shape in a generalist plant is promoted by the preference patterns of its local pollinators. Proc R Soc Lond, Ser B: Biol Sci. 2008;275(1648):2241–9. 10.1098/rspb.2008.0512 . PMC2603243 18544510

[13] Galen C. Regulation of Seed-Set in Polemonium-Viscosum—Floral Scents, Pollination, and Resources. Ecology. 1985;66(3):792–7.

[14] Herrera CM, Castellanos MC, Medrano M. Geographic context of floral evolution: towards an improved research programme in floral diversification In: Harder LD, Barrett SCH, editors. Ecology and evolution of flowers. Oxford: Oxford University Press; 2006 p. 278–94.

[15] Malo JE, Baonza J. Are there predictable clines in plant–pollinator interactions along altitudinal gradients? The example of *Cytisus scoparius* (L.) Link in the Sierra de Guadarrama (Central Spain). Divers Distrib. 2002;8(6):365–71. 10.1046/j.1472-4642.2002.00161.x .

[16] Price MV, Waser NM, Irwin RE, Campbell DR, Brody AK. Temporal and spatial variation in pollination of a montane herb: a seven-year study. Ecology. 2005;86(8):2106–16. 10.1890/04-1274 .

[17] Brunet J. Pollinators of the Rocky Mountain columbine: temporal variation, functional groups and associations with floral traits. Ann Bot. 2009;103(9):1567–78. 10.1093/aob/mcp096 . 19414518 PMC2701757

[18] Bustamante E, Casas A, Búrquez A. Geographic variation in reproductive success of *Stenocereus thurberi* (Cactaceae): Effects of pollination timing and pollinator guild. Am J Bot. 2010;97(12):2020–30. 10.3732/ajb.1000071 . 21616849

[19] Brown M, Downs CT, Johnson SD. Covariation of flower traits and bird pollinator assemblages among populations of *Kniphofia linearifolia* (Asphodelaceae). Plant Syst Evol. 2011;294(3–4):199–206. 10.1007/s00606-011-0443-1 .

[20] Thompson JN, Cunningham BM. Geographic structure and dynamics of coevolutionary selection. Nature. 2002;417(6890):735–8. 10.1038/nature00810 . 12066183

[21] Gómez JM, Perfectti F. Evolution of complex traits: the case of *Erysimum* corolla shape. Int J Plant Sci. 2010;171(9):987–98. 10.1086/656475 .

[22] Thompson JN. The geographic mosaic of coevolution Chicago, London: The University of Chicago Press; 2005 i-xii, 1–443 p.

[23] Thompson JN, Fernandez CC. Temporal dynamics of antagonism and mutualism in a geographically variable plant-insect interaction. Ecology. 2006;87(1):103–12. 10.1890/05-0123 . 16634301

[24] Johnson SD, Steiner KE. Long-tongued fly pollination and evolution of floral spur length in the *Disa draconis* complex (Orcdhidaceae). Evolution. 1997;51(1):45–53. 10.2307/2410959 . 28568792

[25] Hopkins R, Levin DA, Rausher MD. Molecular signatures of selection on reproductive character displacement of flower colour in *Phlox drummondii*. Evolution. 2012;66(2):469–85. 10.1111/j.1558-5646.2011.01452.x . 22276542

[26] Moeller DA. Geographic structure of pollinator communities, reproductive assurance, and the evolution of self-pollination. Ecology. 2006;87(6):1510–22. 10.1890/0012-9658(2006)87[1510:gsopcr]2.0.co;2 . 16869427

[27] Boberg E, Alexandersson R, Jonsson M, Maad J, Ågren J, Nilsson LA. Pollinator shifts and the evolution of spur length in the moth-pollinated orchid Platanthera bifolia. Ann Bot. 2014;113(2):267–75. 10.1093/aob/mct217 . 24169591 PMC3890388

[28] Salzmann CC, Cozzolino S, Schiestl FP. Floral scent in food-deceptive orchids: Species specificity and sources of variability. Plant Biol. 2007;9(6):720–9. 10.1055/s-2007-965614 . 17891704

[29] Suinyuy TN, Donaldson JS, Johnson SD. Geographical variation in cone volatile composition among populations of the African cycad *Encephalartos villosus*. Biol J Linn Soc. 2012;106(3):514–27. 10.1111/j.1095-8312.2012.01905.x .

[30] Majetic CJ, Raguso RA, Ashman T-L. The impact of biochemistry vs. population membership on floral scent profiles in colour polymorphic *Hesperis matronalis*. Ann Bot. 2008;102(6):911–22. 10.1093/aob/mcn181 . 18819948 PMC2712399

[31] Soler C, Hossaert-McKey M, Buatois B, Bessière J-M, Schatz B, Proffit M. Geographic variation of floral scent in a highly specialized pollination mutualism. Phytochemistry. 2011;72(1):74–81. 10.1016/j.phytochem.2010.10.012 . 21109272

[32] Pellmyr O. Three pollination morphs in *Cimicifuga simplex*; incipient speciation due to inferiority competition. Oecologia. 1986;68:304–7. 28310144 10.1007/BF00384804

[33] Huber FK, Kaiser R, Sauter W, Schiestl FP. Floral scent emission and pollinator attraction in two species of *Gymnadenia* (Orchidaceae). Oecologia. 2005;142(4):564–75. 10.1007/s00442-004-1750-9 . 15586296

[34] Junker RR, Blüthgen N. Floral scents repel facultative flower visitors, but attract obligate ones. Ann Bot. 2010;105(5):777–82. 10.1093/aob/mcq045 . 20228087 PMC2859918

[35] Bischoff M, Lord JM, Robertson AW, Dyer AG. Hymenopteran pollinators as agents of selection on flower colour in the New Zealand mountains: salient chromatic signals enhance flower discrimination. New Zealand Journal of Botany. 2013;51(3):181–93. 10.1080/0028825x.2013.806933 .

[36] Chittka L, Raine NE. Recognition of flowers by pollinators. Current Opinion in Plant Biology. 2006;9(4):428–35. . 16713328 10.1016/j.pbi.2006.05.002

[37] Schiestl FP. Ecology and evolution of floral volatile-mediated information transfer in plants. New Phytologist. 2015;206(2):571–7. 10.1111/nph.13243 . 25605223

[38] Caruso CM, Scott SL, Wray JC, Walsh CA. Pollinators, herbivores, and the maintenance of flower color variation: a case study with *Lobelia siphilitica*. Int J Plant Sci. 2010;171(9):1020–8. 10.1086/656511 .

[39] Campbell DR, Bischoff M, Lord JM, Robertson AW. Where have all the blue flowers gone: pollinator responses and selection on flower colour in New Zealand *Wahlenbergia albomarginata*. J Evol Biol. 2012;25(2):352–64. 10.1111/j.1420-9101.2011.02430.x . 22151952

[40] Parachnowitsch AL, Raguso RA, Kessler A. Phenotypic selection to increase floral scent emission, but not flower size or colour in bee-pollinated *Penstemon digitalis*. New Phytol. 2012;195(3):667–75. 10.1111/j.1469-8137.2012.04188.x . 22646058

[41] Hess HE, Landolt E, Hirzel R. Flora der Schweiz und angrenzender Gebiete 2nd, revised edition ed. Basel: Birkhäuser Verlag; 1976.

[42] Fenster CB, Armbruster WS, Wilson P, Dudash MR, Thomson JD. Pollination syndromes and floral specialization. Annual Review of Ecology Evolution and Systematics. 2004;35:375–403. 10.1146/annurev.ecolsys.34.011802.132347 .

[43] van der Cingel NA. An atlas of orchid pollination European orchids. Rotterdam, Netherlands: Balkema; 1995.

[44] Sun M, Gross K, Schiestl FP. Floral adaptation to local pollinator guilds in a terrestrial orchid. Ann Bot. 2014;113:289–300. 10.1093/aob/mct219 24107683 PMC3890390

[45] Hultén E, Fries M. Atlas of North European vascular plants: north of the Tropic of Cancer Königstein: Koeltz Scientific Books,; 1986.

[46] Gustafsson S, Sjögren-Gulve P. Genetic diversity in the rare orchid, *Gymnadenia odoratissima* and a comparison with the more common congener, *G*. *conopsea*. Conserv Genet. 2002;3(3):225–34. 10.1023/a:1019969014333 .

[47] Sletvold N, Ågren J. Pollinator-mediated selection on floral display and spur length in the orchid *Gymnadenia conopsea*. Int J Plant Sci. 2010;171(9):999–1009. 10.1086/656597 .

[48] Bray JR, Curtis JT. An ordination of the upland forest communities of Southern Wisconsin. Ecol Monogr. 1957;27(4):326–49. .

[49] Goslee SC, Urban DL. The ecodist package for dissimilarity-based analysis of ecological data. J Stat Softw. 2007;22(7):1–19.

[50] RCoreTeam. R: A language and environment for statistical computing Vienna, Austria: R Foundation for Statistical Computing; 2013.

[51] Lande R, Arnold SJ. The measurement of selection on correlated characters. Evolution. 1983;37(6):1210–26. 10.2307/2408842 . 28556011

[52] Bates D, Maechler M, Bolker B. lme4: Linear mixed-effects models using S4 classes. 2013;R package version 0.999999–2.

[53] Oksanen JF, Blanchet G, Kindt R, Legendre P, Minchin PR, O'Hara RB, et al. vegan: Community Ecology Package. 2013;R package version 2.0–9.

[54] Sandring S, Ågren J. Pollinator-mediated selection on floral display and flowering time in the perennial herb *Arabidopsis lyrata*. Evolution. 2009;63(5):1292–300. 10.1111/j.1558-5646.2009.00624.x . 19154392

[55] Ehrlén J, Borg-Karlson A-K, Kolb A. Selection on plant optical traits and floral scent: Effects via seed development and antagonistic interactions. Basic Appl Ecol. 2012;13(6):509–15. 10.1016/j.baae.2012.08.001 .

[56] Maad J. Phenotypic selection in hawkmoth-pollinated *Platanthera bifolia*: targets and fitness surfaces. Evolution. 2000;54(1):112–23. 10.1111/j.0014-3820.2000.tb00012.x . 10937188

[57] Sletvold N, Grindeland JM, Ågren J. Pollinator-mediated selection on floral display, spur length and flowering phenology in the deceptive orchid *Dactylorhiza lapponica*. New Phytol. 2010;188(2):385–92. 10.1111/j.1469-8137.2010.03296.x . 20497348

[58] Schiestl FP, Huber FK, Gomez JM. Phenotypic selection on floral scent: trade-off between attraction and deterrence? Evol Ecol. 2011;25(2):237–48. 10.1007/s10682-010-9409-y .

[59] Reynolds RJ, Dudash MR, Fenster CB. Multiyear study of multivariate linear and nonlinear phenotypic selection on floral traits of hummingbird-pollinated *Silene virginia*. Evolution. 2010;64(2):358–69. 10.1111/j.1558-5646.2009.00805.x . 19663992

[60] Dudash MR, Hassler C, Stevens PM, Fenster CB. Experimental floral and inflorescence trait manipulations affect pollinator preference and function in a hummingbird-pollinated plant. Am J Bot. 2011;98(2):275–82. 10.3732/ajb.1000350 . 21613116

[61] Renoult JP, Thomann M, Schaefer HM, Cheptou P-O. Selection on quantitative colour variation in *Centaurea cyanus*: the role of the pollinator's visual system. J Evol Biol. 2013;26(11):2415–27. 10.1111/jeb.12234 24070120

[62] Mant J, Peakall R, Schiestl FP. Does selection on floral odor promote differentiation among populations and species of the sexually deceptive orchid genus *Ophrys*? Evolution. 2005;59(7):1449–63. 10.1111/j.0014-3820.2005.tb01795.x . 16153031

[63] Dötterl S, Wolfe LM, Jürgens A. Qualitative and quantitative analyses of flower scent in *Silene latifolia*. Phytochemistry. 2005;66(2):203–13. 10.1016/j.phytochem.2004.12.002 . 15652577

[64] Siepielski AM, Gotanda KM, Morrissey MB, Diamond SE, Dibattista JD, Carlson SM. The spatial patterns of directional phenotypic selection. Ecol Lett. 2013;16(11):1382–92. 10.1111/ele.12174 . 24028500

[65] Hall MC, Willis JH. Divergent selection on flowering time contributes to local adaptation in *Mimulus guttatus* populations. Evolution. 2006;60(12):2466–77. 10.1554/05-688.1 . 17263109

[66] Zu P, Blanckenhorn WU, Schiestl FP. Heritability of floral volatiles and pleiotropic responses to artificial selection in Brassica rapa. New Phytologist. 2015; 10.1111/nph.13652 26391626

