# Supplementary figures and images for: Why Do Floral Perfumes Become Different? Region-Specific Selection on Floral Scent in a Terrestrial Orchid
Source: PLoS One. 2016 Feb 17;11(2):e0147975. doi: 10.1371/journal.pone.0147975 (PMC4757410; doi:10.1371/journal.pone.0147975)

S1 Fig

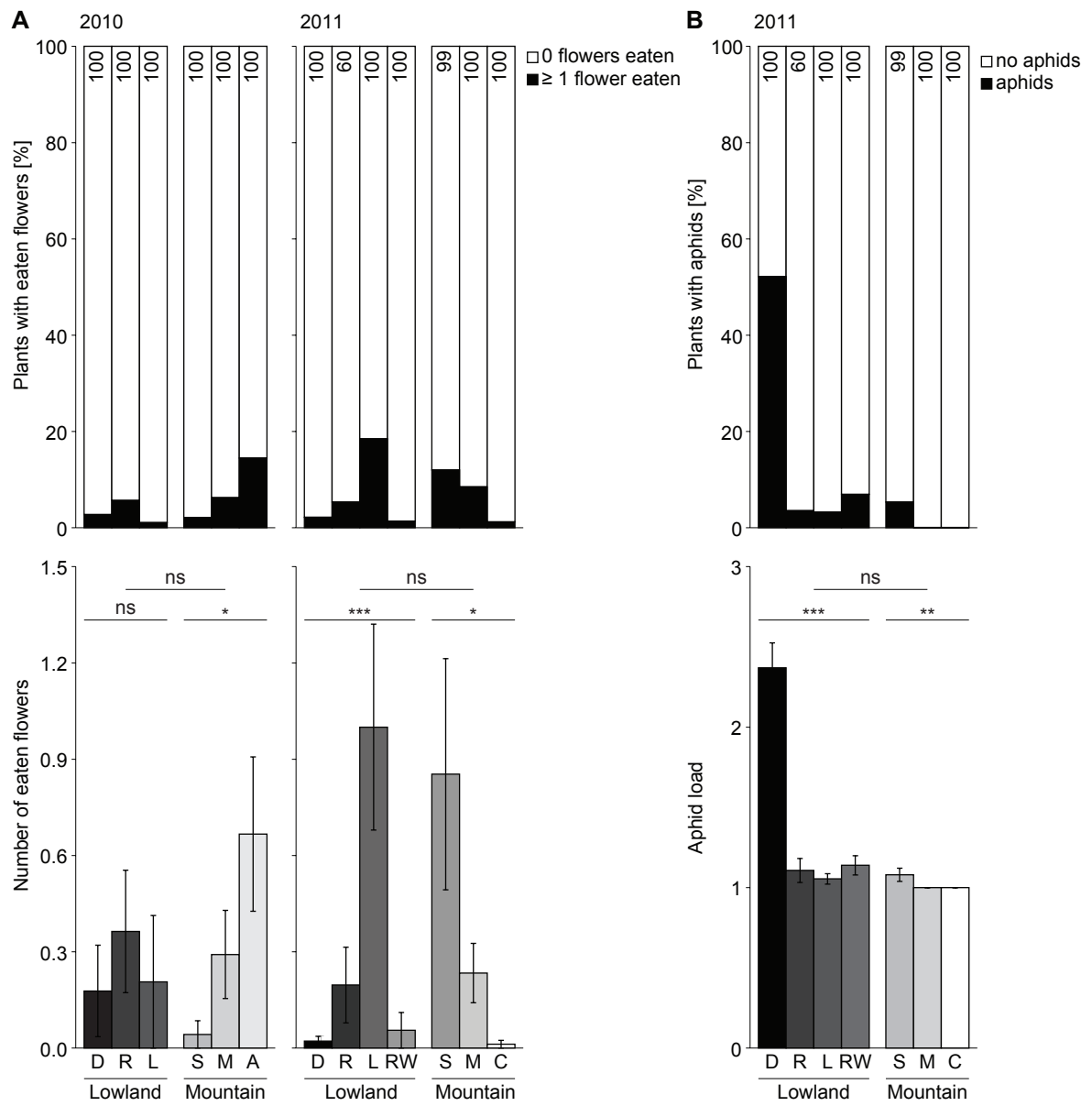

Supplement: S1 Fig — Floral herbivory was quantified as (A) number of eaten flowers per inflorescence and (B) aphid load (scale from 1 [no aphids] to 6 [many aphids]). Sample sizes are indicated inside the top of the percentage bars. Whereas populations within regions differed in floral herbivory, no consistent differences in floral herbivory was found between lowland and mountain regions (***P < 0.001, **P < 0.01, *P < 0.05, ns P > 0.05). (PDF) [file pone.0147975.s001.pdf]

S2 Fig

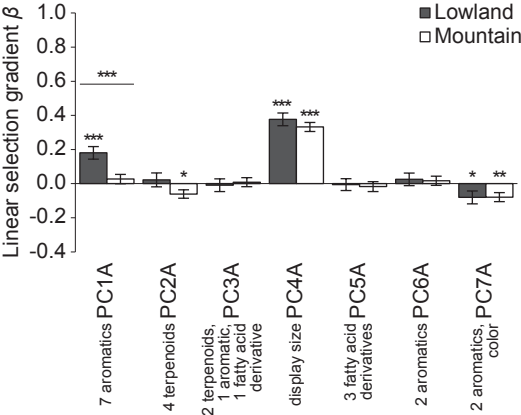

Supplement: S2 Fig — Whereas several PCs showed significant selection gradients (marked with asterisks above bars), only PC1 showed significant differences between the regions (***P < 0.001, **P < 0.01, *P < 0.05). A short description of the floral signals loading primarily on each PC is given; for details, see S4 Table. According to variables loading primarily on PCs, PC1A corresponds to PC1 in Fig 1, PC2A to PC2, PC3A to PC5, PC4A to PC3, PC5A to PC6, PC6A to PC7, and PC7A to PC4 except that floral color additionally loaded primarily on PC7A. nlowland = 312 (four populations), nmountain = 251 (three populations). (PDF) [file pone.0147975.s002.pdf]

S3 Fig

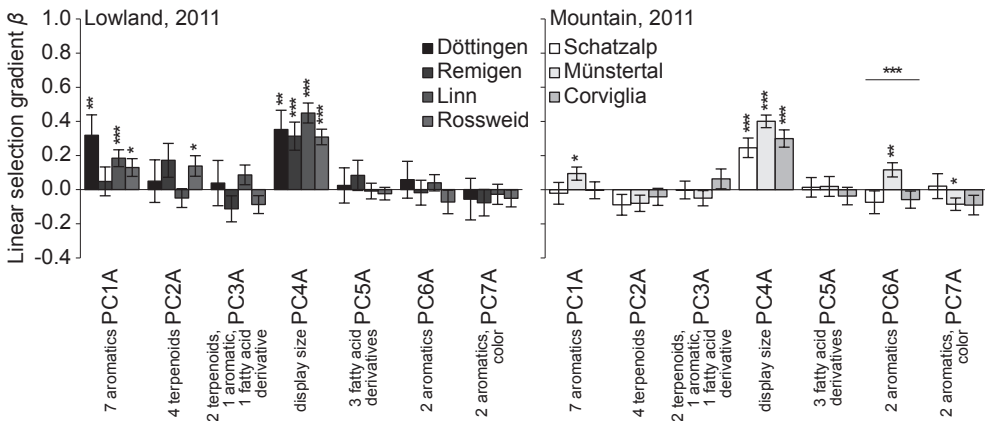

Supplement: S3 Fig — Several PCs showed significant selection gradients and PC6A was significantly different among mountain populations (***P < 0.001, **P < 0.01, *P < 0.05). A short description of the floral signals loading primarily on each PC is given; for details, see S4 Table. According to variables loading primarily on PCs, PC1A corresponds to PC1 in Fig 2, PC2A to PC2, PC3A to PC5, PC4A to PC3, PC5A to PC6, PC6A to PC7, and PC7A to PC4 except that floral color additionally loaded primarily on PC7A. nDöttingen = 92, nRemigen = 56, nLinn = 92, nRossweid = 72, nSchatzalp = 75, nMünstertal = 94, nCorviglia = 82. (PDF) [file pone.0147975.s003.pdf]

**S4 Fig**

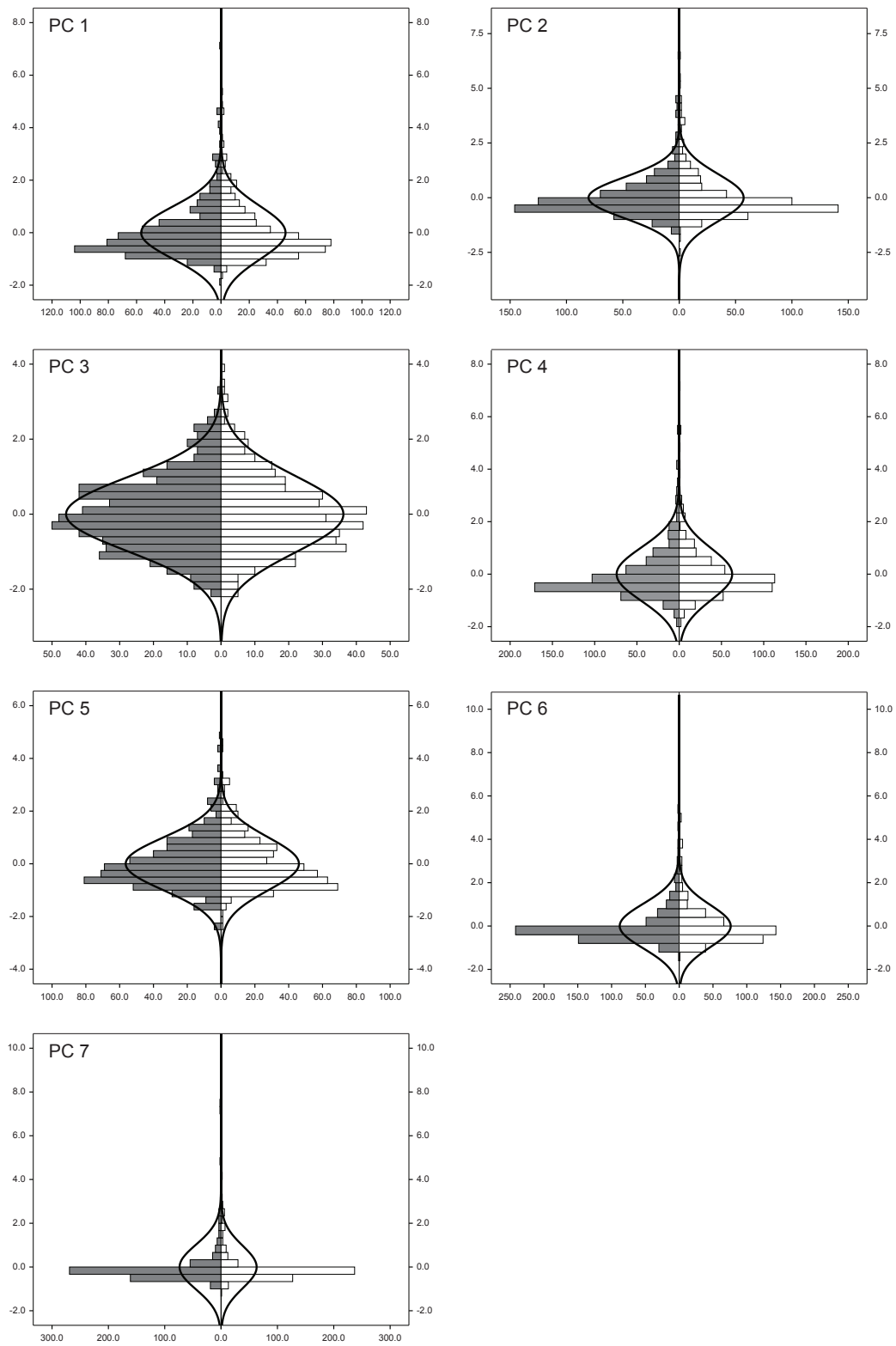

Supplement: S4 Fig — The distributions of the PC scores are compared with superimposed normal distributions. (PDF) [file pone.0147975.s004.pdf]

**S5 Fig**

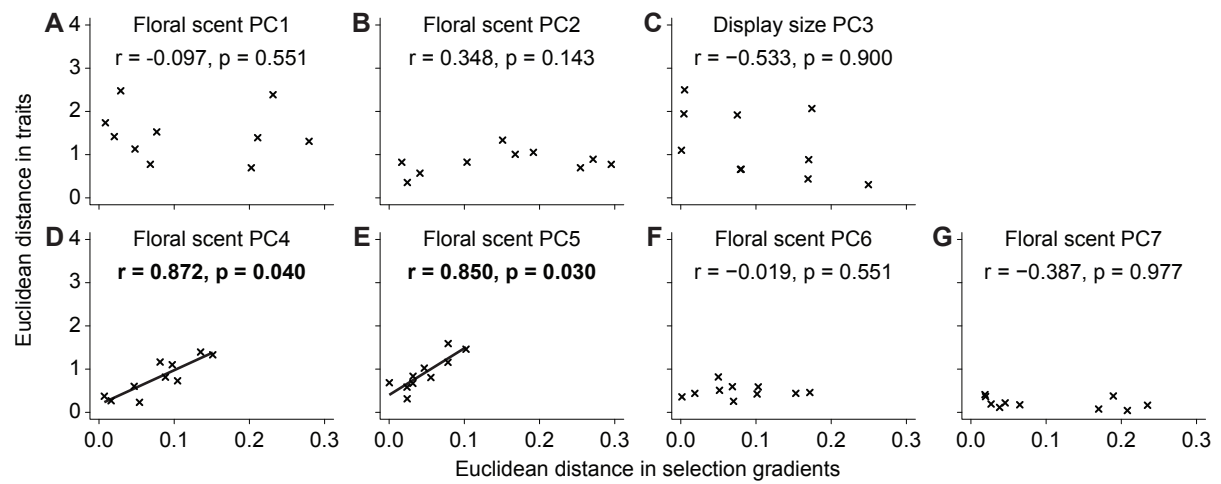

Supplement: S5 Fig — For the Euclidian distances in the signals, only the variables were used that exhibited the highest loadings on the principal components (PCs) used in the selection analysis. For scent PC4 and PC5, a significant association between trait differences and selection differences was found (Mantel test statistics with 1000 permutations in all tests). n = 5 populations (three lowland and two mountain populations). For details on PCs, see Table 1 and S3 Table. (PDF) [file pone.0147975.s005.pdf]
