# Supplementary material for: Why Do Floral Perfumes Become Different? Region-Specific Selection on Floral Scent in a Terrestrial Orchid
Source: PLoS One. 2016 Feb 17;11(2):e0147975. doi: 10.1371/journal.pone.0147975 (PMC4757410; doi:10.1371/journal.pone.0147975)
Supplement: S1 Table — (PDF) [file pone.0147975.s006.pdf]

S1 Table. Geographic locations of the four lowland and the four mountain study-populations of *Gymnadenia odoratissima* and year(s), in which selection and floral signals were measured and hand pollinations were conducted.

Population (code)	Geographic coordinates	Altitude (m a.s.l.)	Selection and floral signals (*n*)	Hand pollination (*n*)
Lowland region				
Döttingen (D)	47°34’30’’N, 08°16’25’’E	500	2010 (100), 2011 (100)	2010 (4), 2011 (9)
Remigen (R)	47°31’45’’N, 08°09’45’’E	600	2010 (100), 2011 (60)	2011 (8)
Linn (L)	47°28’35’’N, 08°07’00’’E	500	2010 (100), 2011 (100)	2011 (9)
Rossweid (RW)	47°18’45’’N, 08°30’40’’E	650	2011 (100)	2011 (10)
Mountain region				
Schatzalp (S)	46°48’20’’N, 09°49’30’’E	1800	2010 (100), 2011 (99)	2010 (5), 2011 (10)
Münstertal (M)	46°37’50’’N, 10°19’05’’E	1800	2010 (100), 2011 (100)	2011 (10)
Albulapass (A)	46°34’55’’N, 09°48’50’’E	2250	2010 (100)	2010 (5)
Corviglia (C)	46°30’20’’N, 09°49’55’’E	2200	2011 (100)	2011 (10)
