# Supplementary material for: Why Do Floral Perfumes Become Different? Region-Specific Selection on Floral Scent in a Terrestrial Orchid
Source: PLoS One. 2016 Feb 17;11(2):e0147975. doi: 10.1371/journal.pone.0147975 (PMC4757410; doi:10.1371/journal.pone.0147975)
Supplement: S2 Table — (PDF) [file pone.0147975.s007.pdf]

S2 Table. Female reproductive success (mean ± SE) of *Gymnadenia odoratissima* plants in the four lowland and the four mountain populations and the statistical tests between lowland populations, between mountain populations, and between the two altitudinal regions.

	Mean ± SE				Statistics	
Trait	Population 1 (*n*)	Population 2 (*n*)	Population 3 (*n*)	Population 4 (*n*)	Test statistic	*P*
Number of fruits per individual						
2010						
Lowland populations	Döttingen (75)	Remigen (91)	Linn (99)			
	15.31 ± 1.91	4.53 ± 0.68	9.64 ± 1.01		F_2 = 21.896	< 0.001
Mountain populations	Schatzalp (47)	Münstertal (99)	Albulapass (85)			
	13.43 ± 1.66	13.44 ± 0.97	9.75 ± 0.96		F_2 = 6.445	0.002
Regions					χ^2_1 = 1.373	0.242
2011						
Lowland populations	Döttingen (96)	Remigen (57)	Linn (100)	Rossweid (95)		
	11.73 ± 1.29	17.21 ± 1.52	17.09 ± 1.07	27.11 ± 1.40	F_3 = 28.574	< 0.001
Mountain populations	Schatzalp (94)	Münstertal (97)		Corviglia (83)		
	16.19 ± 0.86	18.82 ± 1.16		18.57 ± 1.16	F_2 = 0.596	0.552
Regions					χ^2_1 = 0.057	0.811
Proportional female reproductive success						
2010						
Lowland populations	Döttingen (75)	Remigen (91)	Linn (99)			
	18.65 ± 2.00	10.11 ± 1.32	22.80 ± 1.99		F_2 = 15.878	< 0.001
Mountain populations	Schatzalp (47)	Münstertal (99)	Albulapass (85)			
	33.93 ± 3.54	45.73 ± 2.20	26.73 ± 2.30		F_2 = 18.626	< 0.001
Regions					χ^2_1 = 5.081	0.024
2011						

Lowland populations	Döttingen (96)	Remigen (57)	Linn (100)	Rossweid (95)		
	19.20 ± 1.89	35.71 ± 2.83	42.42 ± 2.09	55.96 ± 2.14	$F_3 = 53.18$	< 0.001
Mountain populations	Schatzalp (94)	Münstertal (97)		Corviglia (83)		
	48.38 ± 2.10	56.22 ± 2.58		52.26 ± 2.69	$F_2 = 3.572$	0.029
Regions					$\chi^2_1 = 3.086$	0.079
