# Supplementary material for: Why Do Floral Perfumes Become Different? Region-Specific Selection on Floral Scent in a Terrestrial Orchid
Source: PLoS One. 2016 Feb 17;11(2):e0147975. doi: 10.1371/journal.pone.0147975 (PMC4757410; doi:10.1371/journal.pone.0147975)
Supplement: S3 Table — (PDF) [file pone.0147975.s008.pdf]

S3 Table. Factor loadings of display size and floral scent compounds of *Gymnadenia odoratissima* plants on principal components (PCs) using the two-year data set.

Trait	PC1	PC2	PC3	PC4	PC5	PC6	PC7
Display size							
Plant height	0.097	-0.004	**0.857**	0.003	-0.009	-0.095	0.035
Inflorescence length	0.049	-0.020	**0.881**	0.038	0.044	-0.054	0.028
Number of flowers	0.067	-0.014	**0.822**	0.085	0.025	0.014	-0.030
Floral scent							
Aromatics							
Styrene	0.105	0.445	-0.079	-0.091	**0.447**	0.010	0.184
Benzaldehyde	**0.874**	0.110	0.067	0.289	0.089	0.033	-0.020
Benzyl alcohol	0.336	0.032	0.045	**0.845**	0.157	0.021	0.057
Phenylacetaldehyde	**0.828**	0.054	0.061	0.222	0.005	0.026	-0.065
Phenylethyl alcohol	0.363	0.039	0.061	**0.833**	0.085	0.048	0.005
Benzyl acetate	**0.903**	0.062	0.096	-0.032	0.076	0.055	0.061
1-Phenyl-1,2-propanedione	**0.782**	0.102	0.001	0.237	0.148	0.072	0.038
Phenylethylacetate	**0.858**	0.060	0.118	-0.015	0.089	0.057	0.041
1-Phenyl-2,3-butanedione	**0.851**	0.068	-0.016	0.162	-0.003	0.014	-0.006
Eugenol	**0.566**	0.088	0.055	0.442	0.093	0.033	0.294
Methyl eugenol	0.056	0.013	0.045	0.052	0.036	0.017	**0.880**
Benzyl benzoate	0.007	0.064	-0.015	0.033	0.053	-0.027	**0.871**
Terpenoids							
α-Pinene	0.254	**0.639**	0.085	-0.036	0.108	0.025	-0.027
Sabinene	0.026	**0.787**	-0.054	0.049	0.080	0.068	0.042
β-Pinene	0.041	**0.904**	-0.037	0.021	0.110	0.008	0.013
6-Methyl-5-hepten-2-one	0.103	0.141	0.063	0.164	**0.824**	0.101	-0.035
Limonene	0.025	**0.795**	0.010	0.126	0.067	0.057	0.021
Geranyl acetone	0.079	0.100	-0.025	0.075	**0.858**	0.008	0.059
Fatty acid derivatives							
(Z)-3-Hexen-1-ol	-0.017	-0.006	-0.108	0.076	0.047	**0.890**	-0.002
Heptanal	0.111	0.274	0.244	0.358	**0.430**	0.151	0.061
(Z)-3-Hexenyl acetate	0.064	0.084	-0.128	0.042	-0.013	**0.899**	-0.031
Hexyl acetate	0.249	0.129	0.217	-0.055	0.253	**0.472**	0.041

Note: For each trait, the highest loading is highlighted in bold. The PCs were extracted from a principal component analysis, which was conducted on traits standardized per population to 0 ± 1 (mean ± SD) using varimax rotation. The seven PCs with an eigenvalue > 1 explained 71.8% of the total variance. PC1 explained 21.8% of the total variance, PC2 11.6%, PC3 10.0%, PC4 9.1%, PC5 7.2%, PC6 6.5%, and PC7 5.7%.
