# Supplementary material for: Why Do Floral Perfumes Become Different? Region-Specific Selection on Floral Scent in a Terrestrial Orchid
Source: PLoS One. 2016 Feb 17;11(2):e0147975. doi: 10.1371/journal.pone.0147975 (PMC4757410; doi:10.1371/journal.pone.0147975)
Supplement: S4 Table — (PDF) [file pone.0147975.s009.pdf]

S4 Table. Factor loadings of floral signals of *Gymnadenia odoratissima* on principal components (PCs) using the 2011 data set, which included also floral color.

Trait	PC1A	PC2A	PC3A	PC4A	PC5A	PC6A	PC7A
Display size							
Plant height	0.105	-0.057	0.071	**0.839**	-0.099	0.077	-0.028
Inflorescence length	0.042	-0.021	0.083	**0.896**	-0.074	0.035	0.008
Number of flowers	0.096	0.025	0.014	**0.820**	0.048	-0.043	0.038
Floral color							
Color code	-0.070	-0.007	-0.109	-0.052	0.056	0.028	**0.554**
Floral scent							
Aromatics							
Styrene	0.106	0.429	**0.530**	-0.152	-0.013	0.193	-0.256
Benzaldehyde	**0.913**	0.055	0.168	0.058	0.014	0.005	0.119
Benzyl alcohol	0.451	0.078	0.370	0.101	-0.046	0.134	**0.619**
Phenylacetaldehyde	**0.857**	0.048	0.041	0.062	0.009	-0.048	0.098
Phenylethyl alcohol	0.457	0.092	0.254	0.086	0.012	-0.007	**0.702**
Benzyl acetate	**0.899**	0.052	0.011	0.078	0.039	0.052	-0.089
1-Phenyl-1,2-propanedione	**0.770**	0.088	0.243	0.031	0.069	0.062	0.078
Phenylethylacetate	**0.865**	0.082	0.032	0.099	0.005	-0.008	-0.066
1-Phenyl-2,3-butanedione	**0.860**	0.069	-0.021	-0.011	0.027	0.002	0.032
Eugenol	**0.674**	0.076	0.146	0.065	0.007	0.202	0.316
Methyl eugenol	0.057	0.005	0.062	0.044	0.016	**0.882**	0.028
Benzyl benzoate	0.042	0.086	0.105	0.016	0.013	**0.871**	0.060
Terpenoids							
α-Pinene	0.360	**0.636**	0.172	-0.006	0.038	-0.020	-0.060
Sabinene	0.032	**0.832**	0.140	-0.034	0.107	0.017	0.048
β-Pinene	0.062	**0.928**	0.168	-0.020	0.035	0.052	0.030
6-Methyl-5-hepten-2-one	0.086	0.240	**0.757**	0.114	0.114	-0.030	0.104
Limonene	0.014	**0.828**	0.142	0.031	0.011	0.037	0.078
Geranyl acetone	0.066	0.185	**0.783**	-0.056	0.066	0.118	-0.049
Fatty acid derivatives							
(Z)-3-Hexen-1-ol	-0.028	-0.029	0.107	-0.100	**0.894**	0.048	0.121
Heptanal	0.180	0.112	**0.745**	0.204	0.013	0.064	0.168
(Z)-3-Hexenyl acetate	0.056	0.169	-0.004	-0.072	**0.916**	-0.005	-0.004
Hexyl acetate	0.332	0.067	0.330	0.165	**0.405**	-0.032	-0.135

Note: For each trait, the highest loading is highlighted in bold. The PCs were extracted from a principal component analysis, which was conducted on traits standardized per population to 0 ± 1 (mean ± SD) using varimax rotation. The seven PCs with an eigenvalue > 1 explained 71.8% of the total variance. PC1A explained 20.4% of the total variance, PC2A 11.4%, PC3A 9.5%, PC4A 8.1%, PC5A 8.0%, PC6A 7.6%, and PC7A 6.7%.
