# Supplementary material for: Why Do Floral Perfumes Become Different? Region-Specific Selection on Floral Scent in a Terrestrial Orchid
Source: PLoS One. 2016 Feb 17;11(2):e0147975. doi: 10.1371/journal.pone.0147975 (PMC4757410; doi:10.1371/journal.pone.0147975)
Supplement: S5 Table — (PDF) [file pone.0147975.s010.pdf]

S5 Table. Pollinator limitation in lowland and mountain populations of *Gymnadenia odoratissima* in 2010 and 2011 assessed in a pollination experiment.

		Open-pollinated plants		Hand-pollinated plants		Mann-Whitney U test	
Population	Year	*n*	Median (minimum-maximum)	*n*	Median (minimum-maximum)	*z*	*P*
Lowland region							
Döttingen	2010	75	0.12 (0.00-0.71)	3	0.50 (0.45-0.59)	2.547	0.011
	2011	96	0.13 (0.00-0.71)	9	0.83 (0.38-1.00)	4.699	< 0.001
Remigen	2011	57	0.37 (0.00-0.77)	8	1.00 (0.67-1.00)	4.424	< 0.001
Linn	2011	100	0.44 (0.00-0.84)	7	0.87 (0.43-1.00)	3.755	< 0.001
Rossweid	2011	95	0.59 (0.00-0.86)	8	0.60 (0.40-0.90)	0.536	0.592
Mountain region							
Schatzalp	2010	47	0.33 (0.00-0.84)	5	0.60 (0.50-1.00)	2.546	0.011
	2011	94	0.50 (0.00-0.88)	10	0.78 (0.71-0.93)	4.406	< 0.001
Münstertal	2011	97	0.62 (0.00-0.98)	10	1.00 (0.75-1.00)	4.903	< 0.001
Albulapass	2010	85	0.24 (0.00-0.79)	3	0.30 (0.24-0.76)	1.176	0.240
Corviglia	2011	83	0.58 (0.00-0.90)	9	0.91 (0.56-1.00)	4.107	< 0.001

Note: Shown are sample sizes and the median (minimum-maximum) proportional female reproductive success of open-pollinated control plants and hand-pollinated plants as well as the statistics of the Mann-Whitney U tests.
