# Supplementary material for: Why Do Floral Perfumes Become Different? Region-Specific Selection on Floral Scent in a Terrestrial Orchid
Source: PLoS One. 2016 Feb 17;11(2):e0147975. doi: 10.1371/journal.pone.0147975 (PMC4757410; doi:10.1371/journal.pone.0147975)
Supplement: S6 Table — (PDF) [file pone.0147975.s011.pdf]

S6 Table. Pollinators caught and/or observed on *Gymnadenia odoratissima* inflorescences in the four lowland populations (Döttingen, Remigen, Linn, and Rossweid) and the three mountain populations (Schatzalp, Albulapass, and Corviglia).

Order	Family	Species	No. (No. caught)	No. with pollinaria	Population	Date	Time
Lepidoptera	Elachistidae	*Anchinia grisescens* FREY	4 (2)	0	Corviglia	08 Aug. 2012	17:30-21:40
		Anchinia laureolella H.-S.	3 (1)	0	Corviglia	08 Aug. 2012	17:10-18:52
	Gelechiidae	*Acompsia tripunctella* DENIS & SCHIFF.	2 (1)	0	Corviglia	03 Aug. 2012	11:35-11:36
	Zygaenidae	*Adscita geryon* HBN.	1 (1)	1	Albulapass	28 July 2010	14:00
			1 (1)	1	Corviglia	03 Aug. 2012	09:30
		Adscita spp.	3 (0)	0	Albulapass	23 July 2012	15:33-16:45
			8 (0)	6	Corviglia	03 Aug. 2012	10:17-12:00
			5 (0)	1		08 Aug. 2012	13:16-14:20
		Zygaena exulans HOCHENWARTH	1 (1)	1	Schatzalp	10 July 2012	15:30
		Zygaena transalpina ESP.	1 (1)	0	Corviglia	03 Aug. 2011	13:15
		Zygaena filipendulae L.	1 (1)	0	Corviglia	03 Aug. 2011	14:05
		Zygaena spp.	4 (0)	0	Corviglia	03 Aug. 2012	10:00-10:58
			1 (0)	0		08 Aug. 2012	12:38
	Tortricidae	*Eana argentana* CL.	1 (1)	0	Schatzalp	19 July 2011	10:00
			1 (1)	0	Corviglia	08 Aug. 2012	20:24
	Pterophoridae	*Platyptilia gonodactyla* DENIS & SCHIFF.	1 (0)	0	Schatzalp	24 July 2012	17:09
			1 (0)	0		31 July 2012	08:29
		Stenoptilia spp.	1 (1)	0	Corviglia	08 Aug. 2012	13:00
		Hellinsia osteodactyla Z.	2 (2)	2	Schatzalp	14 July 2010	18:00-18:00
		Pterophoridae spp.	1 (1)	0	Linn	14 July 2011	11:45
			1 (0)	0	Corviglia	03 Aug. 2012	10:53
			12 (0)	2		08 Aug. 2012	14:50-20:54
	Pyralidae	*Endotricha flammealis* DENIS & SCHIFF.	1 (1)	0	Remigen	15 July 2011	21:00
		Oncocera semirubella SCOP.	2 (2)	2	Remigen	15 July 2011	19:00-20:15

		1 (1)	1	Linn	16 July 2011	10:30
	Pempelia palumbella DENIS & SCHIFF.	1 (1)	0	Schatzalp	14 July 2010	22:30
Crambidae	*Eudonia sudetica* Z.	3 (3)	0	Schatzalp	14 July 2010	22:00-22:30
		4 (1)	1		24 July 2012	17:16-18:10
		5 (5)	0		31 July 2012	16:17-22:10
		14 (5)	2	Corviglia	08 Aug. 2012	16:25-22:15
	Crambus lathoniellus ZNCK.	1 (1)	1	Schatzalp	24 July 2012	17:30
	Catoptria radiella HBN.	1 (1)	1	Corviglia	03 Aug. 2012	10:50
	Catoptria conchella DENIS & SCHIFF.	1 (1)	0	Schatzalp	26 July 2012	11:19
		1 (1)	0		31 July 2012	16:05
	Crambus/*Catoptria* spp.	1 (0)	0	Schatzalp	24 July 2012	10:44
		1 (0)	1	Corviglia	08 Aug. 2012	19:33
	Pyrausta despicata L.	1 (1)	1	Döttingen	06 July 2010	range 10:00-14:30
		1 (1)	1		05 July 2012	11:12
Hesperiidae	*Ochlodes sylvanus* ESP.	1 (1)	1	Döttingen	05 July 2012	12:15
		1 (1)	0		07 July 2012	16:05
		1 (1)	0	Remigen	18 July 2012	11:10
		3 (0)	0		23 July 2012	11:55-12:17
		3 (0)	0	Rossweid	09 July 2012	11:15-12:41
		3 (2)	0		13 July 2012	12:30-12:42
Pieridae	*Pieris rapae* L.	1 (1)	0	Döttingen	03 July 2012	11:20
	Pieris napi L.	1 (0)	0	Schatzalp	24 July 2012	09:45
Lycaenidae	*Lycaena tityrus* PODA.	1 (1)	0	Döttingen	03 July 2012	11:40
		1 (1)	0		07 July 2012	16:00
	Plebejus idas L.	1 (1)	1	Albulapass	28 July 2010	14:00
	Plebejus orbitulus PRUN.	1 (1)	1	Corviglia	03 Aug. 2012	11:25
	Polyommatus icarus ROTTEMBURG	2 (0)	0	Corviglia	08 Aug. 2012	11:40-11:55

	Polyommatus eros O.	1 (0)	0	Albulapass	23 July 2012	16:18
		1 (1)	0	Corviglia	03 Aug. 2012	09:40
	Polyommatus coridon PODA.	1 (1)	0	Schatzalp	21 July 2011	12:45
		2 (2)	0		26 July 2012	11:36-11:45
	Polyommatus spp.	5 (0)	0	Schatzalp	26 July 2012	10:50-11:40
		4 (0)	0		01 Aug. 2012	range 09:00-16:00
		3 (0)	0		02 Aug. 2012	16:30-18:00
		2 (0)	0		03 Aug. 2012	07:30-11:45
		1 (0)	0	Albulapass	23 July 2012	16:15
		6 (0)	0	Corviglia	03 Aug. 2012	09:35-11:50
		11 (0)	1		08 Aug. 2012	11:27-15:23
Nymphalidae	*Erebia ligea* L.	1 (1)	0	Schatzalp	26 July 2012	10:40
	Erebia pronoe ESP.	1 (1)	0	Schatzalp	21 July 2011	12:40
	Aphantopus hyperantus L.	1 (1)	0	Döttingen	03 July 2012	12:30
	Maniola jurtina L.	1 (1)	0	Linn	15 July 2011	10:00
		2 (2)	0	Döttingen	07 July 2012	15:30-16:15
		1 (0)	0	Remigen	18 July 2012	17:12
	Argynnis adippe DENIS & SCHIFF.	1 (0)	1	Remigen	18 July 2012	15:46
	Euphydryas aurinia debilis ROTTEMBURG	2 (0)	1	Albulapass	23 July 2012	15:40-16:22
Geometridae	*Elophos dilucidaria* DENIS & SCHIFF.	1 (1)	0	Schatzalp	01 Aug. 2012	12:54
		1 (1)	0	Corviglia	08 Aug. 2012	13:20
	Scopula incanata L.	1 (1)	0	Corviglia	08 Aug. 2012	18:15
	Scopula ternata SCHRANK	1 (1)	0	Schatzalp	14 July 2010	23:05
	Entephtia caesiata DENIS & SCHIFF.	1 (1)	1	Schatzalp	14 July 2010	23:00
		1 (1)	0		31 July 2012	21:30

	Noctuidae	*Phytometra viridaria* CL.	2 (1)	1	Döttingen	29 June 2012	19:20-21:00
			1 (1)	1	Remigen	12 July 2011	11:45
			2 (2)	0		15 July 2011	15:15-17:45
			3 (3)	3		07 July 2012	15:00-17:00
			1 (0)	1		18 July 2012	15:31
Diptera	Empididae	Empididae spp.	1 (1)	0	Schatzalp	15 July 2010	19:20
			1 (1)	0		19 July 2011	10:15
			1 (1)	1		21 July 2011	13:40
			1 (0)	0		24 July 2012	12:00
			9 (3)	0		26 July 2012	10:15-14:57
			1 (1)	0		31 July 2012	16:58
			1 (0)	0		09 Aug. 2012	11:15
			2 (1)	0	Corviglia	08 Aug. 2012	14:35-20:21
Coleoptera		Coleoptera sp. 1	1 (1)	1	Linn	16 July 2011	18:15
Coleoptera		Coleoptera sp. 2	1 (1)	0	Schatzalp	01 Aug. 2012	10:14
