# Supplementary material for: Why Do Floral Perfumes Become Different? Region-Specific Selection on Floral Scent in a Terrestrial Orchid
Source: PLoS One. 2016 Feb 17;11(2):e0147975. doi: 10.1371/journal.pone.0147975 (PMC4757410; doi:10.1371/journal.pone.0147975)
Supplement: S7 Table — (PDF) [file pone.0147975.s012.pdf]

S7 Table. Contribution of pollinator taxa to the differences in the pollinator communities between the lowland and the mountain region as well as between populations within altitudinal regions of *Gymnadenia odoratissima* using SIMPER (Similarity Percentages) analyses.

Pollinator taxa	Average abundance		Contribution (mean ± SD %)	Cumulative contribution [%]
	Lowland region	Mountain region		
Regions				
**Lycaenidae**	0.019	1.015	23.97 ± 4.70	25.43
**Zygaenidae**	0.000	1.039	21.21 ± 16.69	47.93
**Crambidae**	0.019	0.419	11.98 ± 8.77	60.64
**Nymphalidae**	0.070	0.351	7.38 ± 8.46	68.47
**Diptera**	0.000	0.168	6.94 ± 8.80	75.84
Pterophoridae	0.017	0.278	6.28 ± 6.26	82.50
Hesperiidae	0.161	0.000	4.63 ± 5.19	87.41
Noctuidae	0.107	0.000	2.84 ± 4.39	90.43
Elachistidae	0.000	0.121	2.46 ± 3.63	93.03
Geometridae	0.000	0.070	2.37 ± 2.15	95.54
Pyralidae	0.055	0.009	1.53 ± 1.53	97.17
Tortricidae	0.000	0.026	0.77 ± 0.58	97.98
Coleoptera	0.017	0.009	0.72 ± 0.80	98.75
Gelechiidae	0.000	0.035	0.70 ± 1.04	99.49
Pieridae	0.009	0.009	0.48 ± 0.55	100.00
Lowland populations	Döttingen	Remigen		
**Noctuidae**	0.015	0.071	28.11 ± 21.72	33.47
**Hesperiidae**	0.015	0.041	18.26 ± 23.25	55.20
**Nymphalidae**	0.022	0.020	12.40 ± 14.54	69.96
**Pyralidae**	0.000	0.030	8.99 ± 18.49	80.67
Crambidae	0.015	0.000	7.87 ± 11.42	90.04
Lycaenidae	0.015	0.000	5.45 ± 7.28	96.52
Pieridae	0.007	0.000	2.92 ± 6.29	100.00
	Döttingen	Linn		
**Nymphalidae**	0.022	0.023	18.47 ± 21.65	19.85
**Pterophoridae**	0.000	0.023	15.44 ± 23.58	36.45
**Crambidae**	0.015	0.000	10.69 ± 14.20	47.94
**Pyralidae**	0.000	0.023	10.42 ± 15.56	59.14
**Coleoptera**	0.000	0.023	10.42 ± 15.56	70.34
Noctuidae	0.015	0.000	9.23 ± 19.47	80.26
Hesperiidae	0.015	0.000	7.76 ± 10.33	88.60
Lycaenidae	0.015	0.000	6.87 ± 8.90	95.99
Pieridae	0.007	0.000	3.73 ± 7.83	100.00
	Döttingen	Rossweid		
**Hesperiidae**	0.015	0.185	63.70 ± 13.85	70.91

Nymphalidae	0.022	0.000	6.96 ± 9.55	78.66
Crambidae	0.015	0.000	6.21 ± 8.05	85.57
Noctuidae	0.015	0.000	5.73 ± 12.07	91.94
Lycaenidae	0.015	0.000	4.73 ± 6.13	97.21
Pieridae	0.007	0.000	2.51 ± 5.28	100.00
	Remigen	Linn		
**Noctuidae**	0.071	0.000	29.50 ± 22.40	31.29
**Hesperiidae**	0.041	0.000	16.14 ± 25.66	48.41
**Nymphalidae**	0.020	0.023	14.70 ± 18.73	64.01
**Pyralidae**	0.030	0.023	14.66 ± 18.03	79.56
Pterophoridae	0.000	0.023	11.13 ± 17.97	91.37
Coleoptera	0.000	0.023	8.14 ± 12.53	100.00
	Remigen	Rossweid		
**Hesperiidae**	0.041	0.185	43.97 ± 23.91	57.31
**Noctuidae**	0.071	0.000	20.57 ± 15.57	84.13
Pyralidae	0.030	0.000	6.94 ± 14.63	93.18
Nymphalidae	0.020	0.000	5.23 ± 11.03	100.00
	Linn	Rossweid		
**Hesperiidae**	0.000	0.185	67.62 ± 8.04	67.61
**Pterophoridae**	0.023	0.000	9.07 ± 14.04	76.68
Nymphalidae	0.023	0.000	9.07 ± 14.04	85.75
Pyralidae	0.023	0.000	7.13 ± 11.04	92.87
Coleoptera	0.023	0.000	7.13 ± 11.04	100.00
Mountain populations	Schatzalp	Albulapass		
**Zygaenidae**	0.002	1.000	42.67 ± 3.60	44.67
**Lycaenidae**	0.038	0.750	33.90 ± 9.44	80.15
Nymphalidae	0.004	0.500	13.87 ± 13.84	94.67
Crambidae	0.036	0.000	1.90 ± 3.82	96.66
Diptera	0.033	0.000	1.72 ± 3.41	98.46
Geometridae	0.009	0.000	0.49 ± 1.08	98.97
Pterophoridae	0.009	0.000	0.48 ± 1.06	99.47
Torticidae	0.002	0.000	0.14 ± 0.53	99.62
Coleoptera	0.002	0.000	0.13 ± 0.49	99.75
Pyralidae	0.002	0.000	0.12 ± 0.47	99.88
Pieridae	0.002	0.000	0.12 ± 0.46	100.00
	Schatzalp	Corviglia		
**Zygaenidae**	0.002	0.364	35.98 ± 22.28	38.39
**Lycaenidae**	0.038	0.364	20.10 ± 12.00	59.84
**Crambidae**	0.036	0.277	12.50 ± 13.26	73.18
Pterophoridae	0.009	0.242	8.87 ± 9.15	82.65
Diptera	0.033	0.035	5.24 ± 8.39	88.24
Elachistidae	0.000	0.121	3.79 ± 5.44	92.28
Gelechiidae	0.000	0.035	2.42 ± 3.50	94.87

Geometridae	0.009	0.035	2.15 ± 3.41	97.16
Torticidae	0.002	0.017	1.04 ± 2.84	98.28
Nymphalidae	0.004	0.000	0.67 ± 2.52	98.99
Coleoptera	0.002	0.000	0.35 ± 1.70	99.37
Pyralidae	0.002	0.000	0.31 ± 1.43	99.69
Pieridae	0.002	0.000	0.29 ± 1.32	100.00
	Albulapass	Corviglia		
**Zygaenidae**	1.000	0.364	20.36 ± 14.17	32.13
**Lycaenidae**	0.750	0.364	16.36 ± 16.86	57.94
**Nymphalidae**	0.500	0.000	10.64 ± 12.31	74.72
Crambidae	0.000	0.277	5.73 ± 7.97	83.76
Pterophoridae	0.000	0.242	5.04 ± 6.87	91.71
Elachistidae	0.000	0.121	2.41 ± 3.89	95.52
Gelechiidae	0.000	0.035	1.11 ± 1.88	97.28
Geometridae	0.000	0.035	0.69 ± 1.11	98.37
Diptera	0.000	0.035	0.69 ± 1.11	99.46
Torticidae	0.000	0.017	0.34 ± 0.56	100.00

Note: The taxa most important for the differences (together contributing ≥ 70% of the difference) are highlighted in bold.
