# Supplementary material for: Why Do Floral Perfumes Become Different? Region-Specific Selection on Floral Scent in a Terrestrial Orchid
Source: PLoS One. 2016 Feb 17;11(2):e0147975. doi: 10.1371/journal.pone.0147975 (PMC4757410; doi:10.1371/journal.pone.0147975)
Supplement: S8 Table — (PDF) [file pone.0147975.s013.pdf]

S8 Table. Differences in floral traits between lowland and mountain plants.

		Mean ± SE		Statistics		
Traits	Principle component (PC)[a]	Lowland region	Mountain region	Region[b]	Population[c]	Year[d]
Display size						
Plant height [cm]	PC3	31.85 ± 0.42	22.18 ± 0.25	780.1***	430.9***	151.1***
Inflorescence length [cm]	PC3	6.05 ± 0.10	5.37 ± 0.07	72.6***	370.2***	30.8***
Number of flowers	PC3	51.37 ± 0.84	33.90 ± 0.47	365.3***	384.7***	3.4
Floral color						
Color code		4.42 ± 0.04	1.89 ± 0.05	1677.1***	50.8***	
Floral scent [ng/l/inflorescence]						
Benzaldehyde[e]	PC1	370.01 ± 19.22	361.03 ± 20.25	0.4	306.0***	635.9***
Phenylacetaldehyde[e]	PC1	563.39 ± 31.91	428.65 ± 26.81	10.6**	294.5***	233.0***
Benzyl acetate[e]	PC1	174.93 ± 9.14	145.55 ± 8.70	9.1**	180.7***	121.4***
1-Phenyl-1,2-propanedione	PC1	1.45 ± 0.07	1.85 ± 0.09	33.5***	189.1***	325.1***
Phenylethyl acetate[e]	PC1	341.95 ± 17.40	210.20 ± 10.36	19.7***	211.2***	35.9***
1-Phenyl-2,3-butanedione[e]	PC1	19.62 ± 1.31	23.14 ± 1.84	0.2	233.2***	7.3**
Eugenol[e]	PC1	49.56 ± 2.75	14.82 ± 1.09	195.0***	256.8***	6.0*
α-Pinene	PC2	42.29 ± 2.34	55.98 ± 2.29	250.1***	336.6***	669.8***
Sabinene	PC2	74.33 ± 7.70	9.41 ± 0.76	366.2***	964.0***	356.5***
β-Pinene	PC2	21.21 ± 1.37	20.82 ± 1.42	54.5***	559.2***	643.8***
Limonene	PC2	228.36 ± 10.41	494.10 ± 55.20	195.7***	752.6***	155.3***
Benzyl alcohol	PC4	67.93 ± 4.62	41.51 ± 2.79	25.1***	453.6***	65.4***
Phenylethyl alcohol	PC4	135.82 ± 8.12	60.65 ± 5.06	85.9***	350.7***	24.2***
Styrene	PC5	29.49 ± 2.37	33.89 ± 2.33	15.6***	175.0***	5.2*
6-Methyl-5-heptene-2-one	PC5	545.36 ± 26.67	376.66 ± 17.31	24.2***	340.2***	4270.3***
Geranyl acetone	PC5	6.21 ± 0.25	5.46 ± 0.18	3.6	243.6***	134.4***
Heptanal	PC5	17.38 ± 0.60	18.81 ± 0.56	102.8***	429.4***	1602.6***

(Z)-3-Hexen-1-ol	PC6	13.06 ± 1.33	18.08 ± 2.05	53.9***	337.5***	838.7***
(Z)-3-Hexenyl acetate	PC6	193.35 ± 15.41	346.80 ± 33.19	104.9***	493.7***	687.3***
Hexyl acetate	PC6	4.32 ± 0.50	6.22 ± 0.42	141.1***	415.2***	405.2***
Methyl eugenol	PC7	1.47 ± 0.25	0.82 ± 0.22	21.6***	23.7**	16.4***
Benzyl benzoate	PC7	1.19 ± 0.21	0.74 ± 0.26	7.1**	11.4	23.3***
Total scent amount		2902.71 ± 111.38	2675.20 ± 111.19	3.0	310.2***	614.9***

Note: The last three columns refer to effects of the independent variables and show the Wald-Chi^2 values resulting from general linear models.

[a] For details on floral trait loadings on PCs, see S3 Table.

[b] For all traits, df = 1.

[c] For all display size and floral scent traits, df = 6; for floral color, df = 5.

[d] For all traits, df = 1.

[e] Floral scent compounds that have been shown to elicit EAD responses in *G. odoratissima* pollinators (Huber, F. K., R. Kaiser, W. Sauter, and F. P. Schiestl. 2005. Floral scent emission and pollinator attraction in two species of *Gymnadenia* (Orchidaceae). Oecologia 142:564-575.).

* $P < 0.05$.

** $P < 0.01$.

*** $P < 0.001$.
