# Supplementary material for: Why Do Floral Perfumes Become Different? Region-Specific Selection on Floral Scent in a Terrestrial Orchid
Source: PLoS One. 2016 Feb 17;11(2):e0147975. doi: 10.1371/journal.pone.0147975 (PMC4757410; doi:10.1371/journal.pone.0147975)
Supplement: S9 Table — (PDF) [file pone.0147975.s014.pdf]

S9 Table. Median (minimum-maximum) coefficient of variation for the three floral-signal groups of *Gymnadenia odoratissima* plants.

Coefficient of variation	Display size (n = 3)	Floral scent (n = 22)	Floral color (n = 1)
Within population	0.277 (0.213-0.291)^{1}	1.113 (0.436-4.157)^{2}	0.278^{1}
Among population	0.206 (0.194-0.209)^{1}	0.644 (0.352-0.943)^{2}	0.091^{1}

Note: Significant differences are indicated by different superscript numbers. "*n*" refers to the number of traits included in each floral signal group.
