# Supplementary material for: Why Do Floral Perfumes Become Different? Region-Specific Selection on Floral Scent in a Terrestrial Orchid
Source: PLoS One. 2016 Feb 17;11(2):e0147975. doi: 10.1371/journal.pone.0147975 (PMC4757410; doi:10.1371/journal.pone.0147975)
Supplement: S1 Text — (PDF) [file pone.0147975.s015.pdf]

S1 Text

Material and Methods

Measurement of floral herbivory

We quantified the magnitude of floral herbivory for all marked plants on the day we measured floral signals. In both years, we counted the number of flowers eaten by florivores. We observed that *G. odoratissima* inflorescences were infested by a variable number of aphids. Therefore, in 2011, we additionally quantified the aphid load on the inflorescences in six categories with 1 being no aphids and 6 many aphids. Moreover, we computed the proportion of plants with at least one flower eaten and the proportion of plants with aphids on the inflorescence for each population and year.

Statistical analysis

Reproductive success and floral herbivory

Differences in reproductive success and floral herbivory between lowland and mountain plants were analyzed using linear mixed models with altitudinal region as fixed factor and population nested within the altitudinal region as random factor for each year using the package *lme4* [1] in R [2]. Differences in reproductive success and herbivory among populations were analyzed by linear models with population as fixed factor for each year and altitudinal region in R [2]. We square root transformed the number of fruits and arcsine (2011) or arcsine square root (2010) transformed the proportional fRS to improve normality and homogeneity of variances.

Variation in selection and traits

To compare the magnitude of spatial and temporal variation in selection between the two signal groups – display size and floral scent – that were quantified in both study years, coefficients of variation (CVs; standard deviation divided by the mean) of the selection gradients on PCs were used. For each PC, we calculated a value of spatial and a value of temporal variation in selection. To obtain the value of spatial variation in selection, CVs were calculated for each year and altitudinal region using population-specific selection gradients as replicates, and absolute values of these CVs were averaged across years and across regions. Similarly, to obtain the value of temporal variation in selection, CVs were calculated for each population using year-specific selection gradients as replicates, and absolute values of these CVs were averaged across populations within altitudinal regions and then across regions. The CVs of selection on floral scent PCs were compared to the CVs of selection on the floral

display PC using one-sample Wilcoxon signed rank tests. These CV analyses were also conducted separately for the two altitudinal regions. The results were the same for the lowland and the mountain region as well as when both altitudinal regions were combined. Therefore, we only report the results for both altitudinal regions combined.

In addition, to compare within- and among-population trait variation between the three floral signal groups display size, floral scent, and floral color, CVs were calculated for each trait within and between populations by dividing within- and among-population SD by each respective mean. Means of CVs were calculated between years and altitudinal regions. We analyzed the differences in the CVs between floral signal groups using Mann-Whitney U tests (display size versus floral scent) or one-sample Wilcoxon signed rank tests (display size and floral size versus floral color).

Correlations between differences in floral signals and differences in selection

To assess correlations between differences in floral signals and differences in selection gradients on these signals, Mantel tests with 1000 permutations were conducted using the package *vegan* (version 2.0-9; [3]) in R [2]. Floral-signal differences were calculated as Euclidean distances between population means (means of the 2010 and 2011 floral signal values) for display size (plant height, inflorescence length, number of flowers), floral color (color intensity code), overall floral scent (all 22 floral scent compounds), and each group of floral scent compounds that exhibited the highest loadings on individual PCs (S3 Table). Data standardized (mean ± SD of 0 ± 1) for each year were used for the Euclidean distance calculations. Similarly, selection gradient differences were calculated as Euclidean distances between population means of selection gradients (means of the 2010 and the 2011 selection gradients) for each PC. We included only populations we measured in both years; these were the lowland populations Döttingen, Remigen, and Linn and the mountain populations Schatzalp and Münstertal. All Euclidean distances were calculated using the package *ecodist* (version 1.2.7; [4]) in R [2].

Results

Variation in selection

Selection on floral scent was consistently more variable than selection on display size. For among-population variation in selection, coefficients of variation (CVs) for selection gradients on floral scent PCs ranged from 0.78 to 13.58 (median = 3.53), which was significantly higher than the CVs for selection gradients on the display size PC (0.21; n = 6, standardized test statistics = 2.201, P = 0.028). Similarly, for temporal variation in selection, the CVs for selection gradients on floral scent PCs ranged from 0.42 to 8.89 (median = 3.12), which was significantly higher than the CVs for selection gradients on the display size PC (0.22; n = 6, standardized test statistics = 2.201, P = 0.028).

Association between selection and floral signals

Floral scent was significantly more variable both within and between populations compared with floral color and display size (S9 Table). We found a high correlation between Euclidean distances in floral signals and selection gradients for some floral scent PCs (S5 Fig), even though it was significant only for floral scent PC4 and floral scent PC5 (S5 Fig D and E).
This shows that the degree of population differences in selection corresponded to differences in the respective scent compound groups. Such a correlation was not detected for display size.

References

1. Bates D, Maechler M, Bolker B. lme4: Linear mixed-effects models using S4 classes. 2013;R package version 0.999999-2.
2. RCoreTeam. R: A language and environment for statistical computing. Vienna, Austria: R Foundation for Statistical Computing; 2013.
3. Oksanen JF, Blanchet G, Kindt R, Legendre P, Minchin PR, O'Hara RB, et al. vegan: Community Ecology Package. 2013;R package version 2.0-9.
4. Goslee SC, Urban DL. The ecodist package for dissimilarity-based analysis of ecological data. J Stat Softw. 2007;22(7):1-19.
